# Videolaryngoscopy versus Fiberoptic Bronchoscopy for Awake Tracheal Intubation: A Systematic Review and Meta-Analysis of Randomized Controlled Trials

**DOI:** 10.3390/jcm13113186

**Published:** 2024-05-29

**Authors:** Raffaele Merola, Maria Vargas, Annachiara Marra, Pasquale Buonanno, Antonio Coviello, Giuseppe Servillo, Carmine Iacovazzo

**Affiliations:** Anesthesia and Intensive Care Medicine, Department of Neurosciences, Reproductive and Odontostomatological Sciences, University of Naples “Federico II”, Via Pansini 5, 80100 Naples, Italy; vargas.maria82@gmail.com (M.V.); dottmarraannachiara@gmail.com (A.M.); pasqual3.buonanno@gmail.com (P.B.); antonio.coviello@unina.it (A.C.); maria.vargas@unina.it (G.S.); carmine.iacovazzo@unina.it (C.I.)

**Keywords:** airway management, awake intubation, fiberoptic bronchoscopy, meta-analysis, trial sequential analysis, videolaryngoscopy

## Abstract

**Background**: In recent years, videolaryngoscopy has increasingly been utilized as an alternative to fiberoptic bronchoscopy in awake intubation. Nonetheless, it remains uncertain whether videolaryngoscopy represents a viable substitute for fiberoptic bronchoscopy. We conducted this systematic review with a meta-analysis to compare videolaryngoscopy and fiberoptic bronchoscopy for awake intubation. **Methods**: We systematically searched for all randomized controlled trials (RCTs) comparing videolaryngoscopy and fiberoptic bronchoscopy for awake intubation. The Cochrane Central Register of Controlled Trials (CENTRAL), Embase, and MEDLINE were systematically queried through August 2023. Our primary outcome measure was the duration of intubation. Secondary outcomes encompassed the rate of successful intubation on the initial attempt, failed intubation, patient-reported satisfaction, and any complications or adverse events potentially stemming from the intubation procedure. The Cochrane Risk of Bias Tool for RCTs was employed to evaluate all studies for evidence of bias. The GRADE approach was utilized to gauge the certainty of the evidence. **Results**: Eleven trials involving 873 patients were ultimately included in our review for data extraction. Meta-analysis demonstrated that videolaryngoscopy decreased the duration of intubation compared to fiberoptic bronchoscopy (SMD −1.9671 [95% CI: −2.7794 to −1.1548] *p* < 0.0001), a finding corroborated in subgroup analysis by the type of videolaryngoscope (SMD −2.5027 [95% CI: −4.8733 to −0.1322] *p* = 0.0385). Additionally, videolaryngoscopy marginally lowered the risk of experiencing a saturation below 90% during the procedure (RR −0.7040 [95% CI: −1.4038 to −0.0043] *p* = 0.0486). No statistically significant disparities were observed between the two techniques in terms of failed intubation, initial successful intubation attempt, or sore throat/hoarseness. With regard to patient-reported satisfaction, a pooled analysis was precluded due to the variability in evaluation methods employed across the trials to assess this outcome. Lastly, trial sequential analysis (TSA) conducted for intubation time (primary outcome) affirmed the conclusiveness of this evidence; TSA performed for secondary outcomes failed to yield conclusive evidence, indicating the necessity for further trials. **Conclusions**: Videolaryngoscopy for awake tracheal intubation diminishes intubation time and the risk of experiencing a saturation below 90% compared to fiberoptic bronchoscopy.

## 1. Introduction

Awake fiberoptic intubation is recommended for managing a known or anticipated difficult airway [1,2]. This technique ensures patient safety by maintaining spontaneous ventilation and intrinsic airway tone until tracheal intubation, thus mitigating the risks associated with managing a difficult airway in a patient under anesthesia [3,4,5,6]. However, learning fiberoptic intubation can be challenging and requires regular practice to maintain proficiency [7,8,9]. Additionally, it carries the potential for various complications, such as oversedation and airway obstruction [10,11,12]. Performing the procedure also demands familiarity with equipment, knowledge of endoscopic airway anatomy, and skill in administering effective local anesthesia and sedation [13]. These drawbacks have contributed to the underutilization of fiberoptic intubation by many anesthesiologists and prompted the exploration of alternative airway devices that offer greater manageability and comparable efficacy.

Recently, videolaryngoscopes (VLS) have seen a surge in usage for managing difficult airways [14,15,16]. VLSs offer improved glottic visualization and reduce the need for repeated laryngoscopy attempts that fail to visualize the glottis, making them valuable for patients with known or anticipated difficult airways [17]. Moreover, they appear to be simpler to use and quicker to master compared to fiberoptic bronchoscopy [18]. Due to these attributes, they are increasingly being employed as an alternative to fiberoptic bronchoscopy for awake intubation. However, given that awake intubation with videolaryngoscopy is a relatively recent technique, it remains unclear whether VLSs represent a viable substitute for fiberoptic bronchoscopy.

We undertook this systematic review with meta-analysis to juxtapose videolaryngoscopy and fiber-optic bronchoscopy for awake intubation. Our primary focus was on intubation time. Secondary endpoints encompassed the success rate of intubation on the initial attempts, instances of failed intubation, patient-reported satisfaction, and any complications or adverse events possibly stemming from the intubation procedure.

## 2. Methods

### 2.1. Search Strategy

This systematic review and meta-analysis were conducted in accordance with the most recent PRISMA guidelines [19]. This study was not registered in any database. We conducted a comprehensive search for all randomized controlled trials (RCTs) comparing videolaryngoscopy and fiberoptic bronchoscopy for awake intubation. We searched the following electronic databases up to August 2023: Cochrane Central Register of Controlled Trials (CENTRAL), Embase, and MEDLINE. The search terms utilized to identify relevant publications included “videolaryngoscopy” OR “videolaryngoscope” AND “awake intubation” AND “randomized controlled trial”. No language restrictions were applied. Non-randomized controlled trials were excluded. The participants comprised patients of all ages and genders requiring tracheal intubation, irrespective of the type of surgery.

### 2.2. Data Extraction and Quality Assessment

Two reviewers (RM and MV) autonomously conducted an initial selection by screening titles and abstracts. Both reviewers examined citations to identify additional RCTs suitable for inclusion in our analysis. Full copies of all potentially relevant studies were procured for thorough evaluation. Data from each study were independently extracted by two reviewers (RM and MV) utilizing standardized spreadsheets in Microsoft Excel (Microsoft Corporation, Redmond, WA, USA), and subsequently cross-verified for discrepancies by a third reviewer (GS). Two reviewers (CI, AM) independently evaluated the study’s quality. Any disagreements were resolved through consensus, with recourse to a pair of reviewers (PB and AC) if necessary. The risk of bias was evaluated using the Cochrane Risk of Bias Tool for RCTs.

### 2.3. Outcome Measures

The primary outcome assessed was the intubation time. Secondary outcomes encompassed the rate of successful intubation on the first attempts, instances of failed intubation, patient-reported satisfaction, and any complications or adverse events potentially arising from the intubation procedure.

### 2.4. Quantitative Analysis

The analysis employed the logarithmic hazard ratio and standardized mean difference as outcome measures. A random effects model was applied to the data. The DerSimonian–Laird estimator was utilized to estimate the amount of heterogeneity (tau^2^). Additionally, the Q-test for heterogeneity and the I^2^ statistic were calculated. If any level of heterogeneity was detected (i.e., tau^2^ > 0, irrespective of the Q-test results), a prediction interval for the true results was provided. Studentized residuals and Cook’s distances were examined to identify potential outliers and influential studies within the model framework. Studies with studentized residuals exceeding the 100 × (1 − 0.05/(2 × k))th percentile of a standard normal distribution were deemed potential outliers (e.g., utilizing a Bonferroni correction with two-sided alpha = 0.05 for k studies included in the meta-analysis). Studies with Cook’s distances surpassing the median plus six times the interquartile range of Cook’s distances were considered influential. The rank correlation test and regression test were employed to assess the asymmetry of the funnel plot, utilizing the standard error of the observed outcomes as a predictor.

Trial sequential analysis (TSA) relies on determining the required information size (RIS), also known as the optimal information size. TSA was conducted using TSA 0.9 beta software when the number of included studies exceeded five. RIS was estimated considering relative risk reduction and information size adjusted for heterogeneity for dichotomous outcomes. A positive result was confirmed if the cumulative Z curve surpassed the sequential monitoring limit of the Lan–DeMets study or reached RIS above the conventional significance level line (Z = 1.96). Conversely, a negative result was confirmed if the cumulative Z curve reached the futility limit or fell below RIS under the conventional significance level line (Z = 1.96). Additionally, 95% CIs adjusted for TSA were provided.

## 3. Results

**Figure 1:**
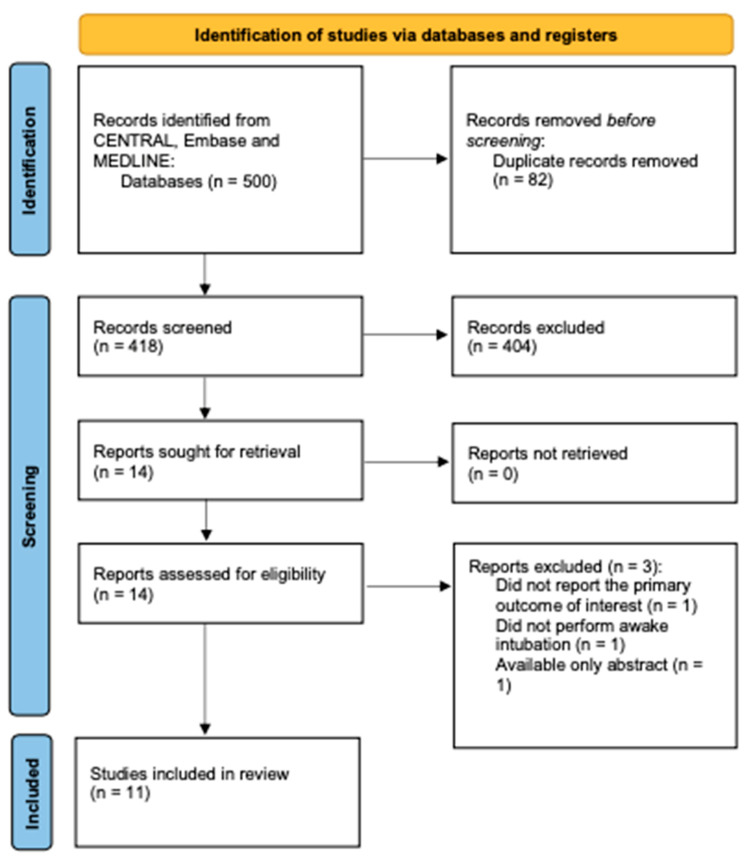
PRISMA 2020 flow diagram for new systematic reviews.

The electronic search yielded 500 potential articles. Following the removal of duplicates, we assessed the eligibility of 418 articles based on title and abstract. Only fourteen studies were selected for full-text evaluation. Three trials were excluded as they did not meet our inclusion criteria. Ultimately, eleven trials, involving 873 patients, were included in our review [20,21,22,23,24,25,26,27,28,29,30]. The screening process is outlined in Figure 1. One study was excluded from the meta-analysis due to incomplete reported data [27]. Baseline characteristics of the included studies are presented in Table 1. Table 2 provides a summary of the risk of bias for each included study.

### 3.1. Duration of Intubation (Time)

**Figure 2:**
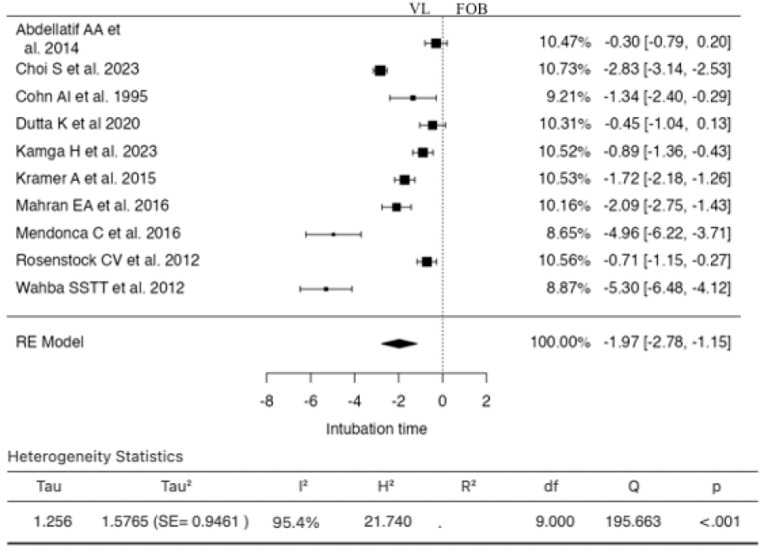
Forest plot for the comparison of intubation time between videolaryngoscopy and fiberoptic bronchoscopy groups.

Ten studies were included in the analysis (Figure 2). The estimated SMD based on the random-effects model was −1.9671 (95% CI: −2.7794 to −1.1548). Thus, the average outcome significantly differed from zero (z = −4.7464, *p* < 0.0001). As per the Q-test, the true outcomes appeared to be heterogeneous (I^2^ = 95.4003%). Examination of the studentized residuals indicated that none of the studies had a value larger than ±2.8070, suggesting no outliers in this model. According to Cook’s distances, none of the studies were considered overly influential. The regression test revealed funnel plot asymmetry (*p* = 0.0102), while the rank correlation test did not show significance (*p* = 0.4843).

### 3.2. Duration of Intubation (Time) with Glidescope

**Figure 3:**
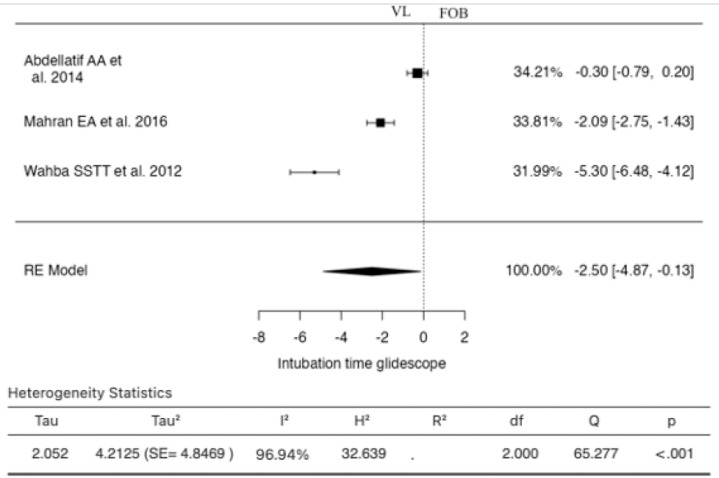
Forest plot for the comparison of intubation time between Glidescope videolaryngoscopy and fiberoptic bronchoscopy groups.

Three studies were included in the analysis for this outcome (Figure 3). The estimated SMD based on the random-effects model was −2.5027 (95% CI: −4.8733 to −0.1322). Thus, the mean result significantly differed from zero (z = −2.0693, *p* = 0.0385). According to the Q-test, the true results appeared to be heterogeneous (I^2^ = 96.9362%). Examination of the studentized residuals revealed that one study (Wahba SSTT et al., 2012) [30] had a value greater than ±2.3940, indicating it could be a potential outlier in this model. According to Cook’s distances, none of the studies were deemed overly influential. The regression test indicated funnel plot skewness (*p* < 0.0001), while the rank correlation test did not show significance (*p* = 0.3333).

### 3.3. Duration of Intubation (Time) with Others VLSs

**Figure 4:**
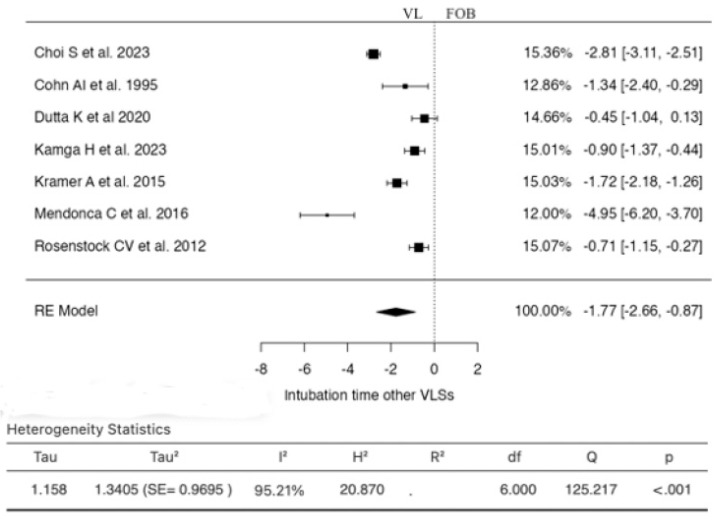
Forest plot for the comparison of intubation time between others videolaryngoscopes and fiberoptic bronchoscopy groups.

Seven studies were included in the analysis for this outcome (Figure 4). The SMD based on the random-effects model was −1.7662 (95% CI: −2.6636 to −0.8688).

In this case, the mean result significantly differed from zero (z = −3.8574, *p* = 0.0001). According to the Q-test, the true results appeared to be heterogeneous (I^2^ = 95.2083%). Examination of the student residuals indicated that one study (Mendonca C et al., 2016) had a value greater than ±2.6901, suggesting it could be a potential outlier in this model. According to Cook’s distances, none of the studies were considered overly influential. Neither the rank correlation test nor the regression test indicated any funnel plot skewness (*p* = 1.0000 and *p* = 0.1421, respectively).

### 3.4. Failed Intubation

**Figure 5:**
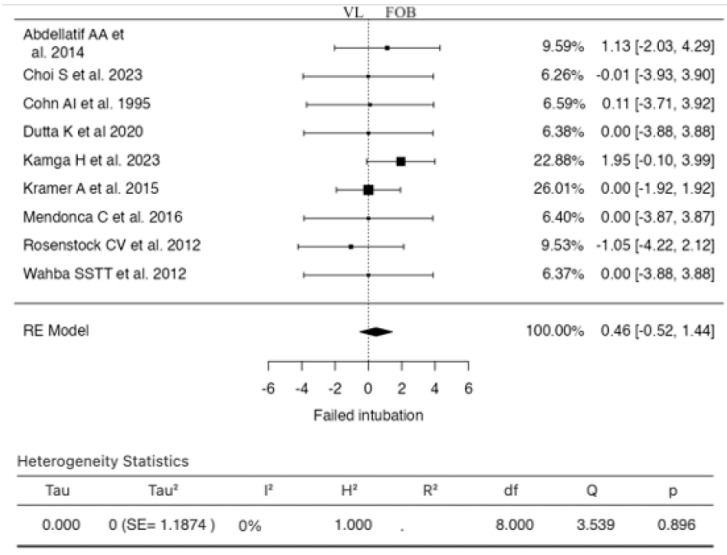
Forest plot for the comparison of failed intubation between videolaryngoscopy and fiberoptic bronchoscopy groups.

Nine studies were included in the analysis for this outcome (Figure 5). The RR based on the random-effects model was 0.4594 (95% CI: −0.5201 to 1.4388). Therefore, the mean result did not differ significantly from zero (z = 0.9192, *p* = 0.3580). According to the Q-test, no significant heterogeneity was found in the true results (I^2^ = 0.0000%). Examination of the student residuals indicated that none of the studies had a value greater than ±2.7729, suggesting no outliers in this model. According to Cook’s distances, one study (Kamga H et al., 2023) could be considered overly influential. Neither the rank correlation test nor the regression test indicated any funnel plot skewness (*p* = 0.6122 and *p* = 0.4346, respectively).

### 3.5. First Attempt Successful Intubation

**Figure 6:**
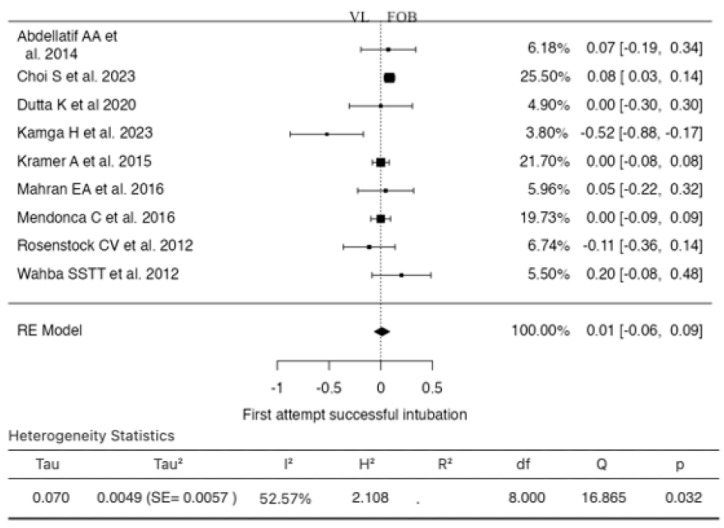
Forest plot for the comparison of first attempt successful intubation between videolaryngoscopy and fiberoptic bronchoscopy groups.

Nine studies were included in the analysis for this outcome (Figure 6). The RR based on the random-effects model was 0.0123 (95% CI: −0.0616 to 0.0863). In this instance, the mean result did not significantly differ from zero (z = 0.3272, *p* = 0.7435). According to the Q-test, the true results appeared to be heterogeneous (I^2^ = 52.5650%). Examination of the studentized residuals revealed that one study (Kamga H et al., 2023) had a higher value of ±2.7729, suggesting it could be a potential outlier in this model. According to Cook’s distances, one study (Kamga H et al., 2023) could be considered overly influential. Neither the rank correlation test nor the regression test indicated any funnel plot skewness (*p* = 0.6122 and *p* = 0.2255, respectively).

### 3.6. Oxygen Saturation Lower Than 90%

**Figure 7:**
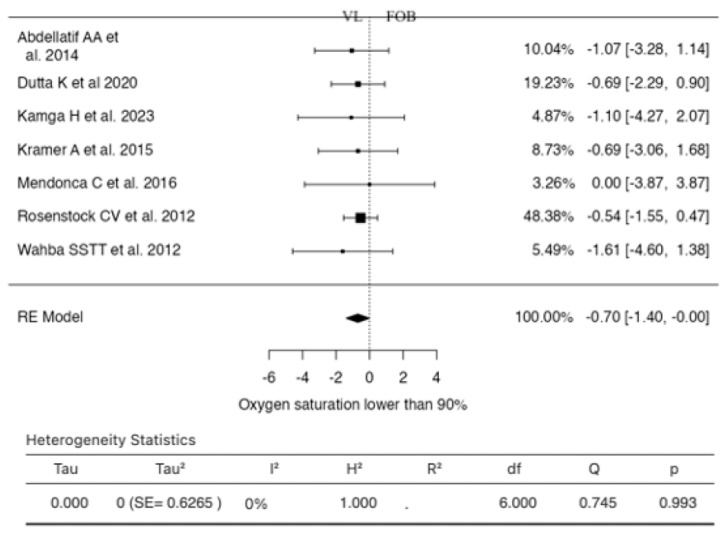
Forest plot for the comparison of oxygen saturation lower than 90% between videolaryngoscopy and fiberoptic bronchoscopy groups.

Seven studies were included in the analysis for this outcome (Figure 7). The RR based on the random-effects model was −0.7040 (95% CI: −1.4038 to −0.0043).

In this scenario, the mean result significantly differed from zero (z = −1.9719, *p* = 0.0486). According to the Q-test, no significant heterogeneity was found in the actual results (I^2^ = 0.0000%). One study (Rosenstock CV et al., 2012) had a relatively high weight compared to the rest of the studies. Examination of the student residuals revealed that none of the studies had a value greater than ±2.6901, indicating no outliers in this model. According to Cook’s distances, one study (Rosenstock CV et al., 2012) could be considered overly influential. Neither the rank correlation test nor the regression test indicated any funnel plot skewness (*p* = 0.5619 and *p* = 0.6883, respectively).

### 3.7. Sore Throat/Hoarseness

**Figure 8:**
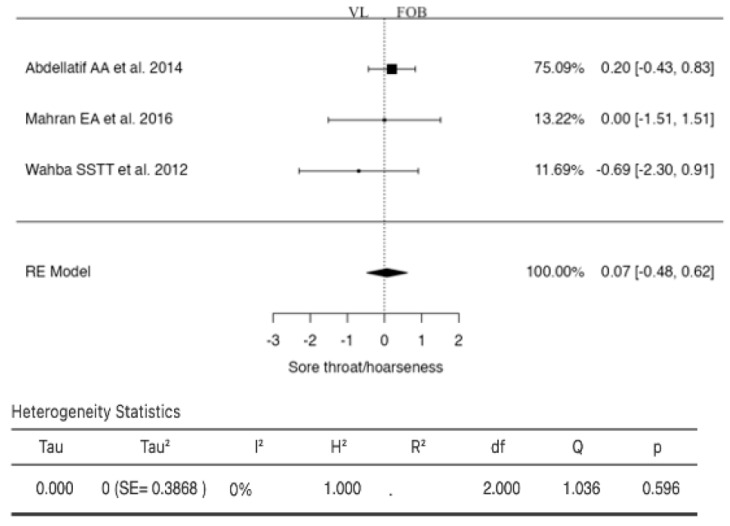
Forest plot for the comparison of sore throat/hoarseness between videolaryngoscopy and fiberoptic bronchoscopy groups.

Three studies were included in the analysis for this outcome (Figure 8). The RR based on the random-effects model was 0.0682 (95% CI: −0.4803 to 0.6168). In this case, the mean result did not significantly differ from zero (z = 0.2438, *p* = 0.8074). According to the Q-test, no significant heterogeneity was found in the true results (I^2^ = 0.0000%). Examination of the student residuals revealed that none of the studies had a value greater than ±2.3940, indicating no outliers in this model. According to Cook’s distances, none of the studies were considered overly influential. Neither the rank correlation test nor the regression test indicated any funnel plot skewness (*p* = 0.3333 and *p* = 0.3980, respectively).

### 3.8. Patient-Reported Satisfaction

Patient satisfaction was reported in seven studies. Abdellatif et al. reported that 58% and 59% of patients rated their experience as excellent in the videolaryngoscopy group and the fiberoptic group, respectively (*p* = 0.92). Similarly, Wahba et al. found that 72% of participants described the procedure as excellent in the videolaryngoscopy group, compared with 64% in the fiberoptic bronchoscopy group (*p* > 0.05). In the study by Mendonca et al., the median visual analog scale (VAS) for patient comfort was 79 [range 59–100] in the videolaryngoscopy group compared to 86 [range 69–100] in the fiberoptic bronchoscopy group (*p* = 0.0616). Rosenstock et al. reported an equal level of patient discomfort during the procedure in both groups, with a median VAS of 2 [range 0–10] for the videolaryngoscopy group and 2 [range 0–6] for the fiberoptic bronchoscopy group (*p* = 0.55). Moore and colleagues stated that there was no difference in patient satisfaction between the groups, but no further information was available. In Kamga and colleagues’ study, the median VAS for patient comfort was 8 [range 2–10] in the videolaryngoscopy group and 8 [range 3–10] for the fiberoptic bronchoscopy group, *p* = 0.370. Finally, Dutta and colleagues in their study found that patient satisfaction was similar with both techniques (*p* = 1.000). An aggregate analysis could not be performed because of the variability in the assessment methods used in the various studies to examine this result.

### 3.9. Sedation with and without Target on Ramsay Score

Sedation with Remifentail infusion targeted for a Ramsay score of 2/3 was used in five studies [20,24,26,27,29] while the other studies did not use a targeted sedation [21,22,23,25,30]. The analysis performed by dividing studies according to targeted and non-targeted sedation did not show any statistically significant results (Supplementary Materials). However, the intubation time was much lower in studies that did not use a targeted sedation Ramsay score than in studies that used it. (Supplementary Materials).

### 3.10. Airway Anticipated to Be Normal

Intubation time: seven studies were included in the analysis. The observed standardized mean differences ranged from −4.9647 to −0.2958, with the majority of estimates being negative (100%). The estimated average standardized mean difference based on the random-effects model was $\hat{\mu }$ = −1.44 (95% CI: −2.1651 to −0.7293). Therefore, the average outcome differed significantly from zero (z = −3.9511, *p* < 0.0001). According to the Q-test, the true outcomes appear to be heterogeneous (Q(6) = 70.7092, *p* < 0.0001, tau^2^ = 0.8316, I^2^ = 91.5145%). A 95% prediction interval for the true outcomes is described by the range of −3.3734 to 0.4789. Hence, although the average outcome is estimated to be negative, in some studies the true outcome may in fact be positive. An examination of the studentized residuals revealed that one study (Mendonca C et al., 2016) had a value larger than ±2.6901 and may be a potential outlier in the context of this model. According to the Cook’s distances, one study (Mendonca C et al., 2016) could be considered to be overly influential. The regression test indicated funnel plot asymmetry (*p* < 0.0001) but not the rank correlation test (*p* = 0.3813) [Supplementary Materials].

First attempt successful intubation: six studies were included in the analysis. The observed log risk ratios ranged from −0.5213 to 0.0726, with the majority of estimates being negative (33%). The estimated average log risk ratio based on the random-effects model was $\hat{\mu }$ = −0.0328 (95% CI: −0.1281 to 0.0626). Therefore, the average outcome did not differ significantly from zero (z = −0.6732, *p* = 0.5008). The Q-test for heterogeneity was not significant, but some heterogeneity may still be present in the true outcomes (Q(5) = 9.2855, *p* = 0.0982, tau^2^ = 0.0055, I^2^ = 46.1527%). A 95% prediction interval for the true outcomes is described by the range −0.2066 to 0.1411. Hence, although the average outcome is estimated to be negative, in some studies the true outcome may in fact be positive. An examination of the studentized residuals revealed that one study (Kamga H et al., 2023) had a value larger than ±2.6383 and may be a potential outlier in the context of this model. According to the Cook’s distances, none of the studies could be considered to be overly influential. Neither the rank correlation nor the regression test indicated any funnel plot asymmetry (*p* = 0.4694 and *p* = 0.1695, respectively) [Supplementary Materials].

Failed intubation: six studies were included in the analysis. The observed log risk ratios ranged from −1.0521 to 1.9459, with the majority of estimates being negative (17%). The estimated average log risk ratio based on the random-effects model was $\hat{\mu }$ = 0.5610 (95% CI: −0.5288 to 1.6508). Therefore, the average outcome did not differ significantly from zero (z = 1.0090, *p* = 0.3130). According to the Q-test, there was no significant amount of heterogeneity in the true outcomes (Q(5) = 3.3630, *p* = 0.6442, tau^2^ = 0.0000, I^2^ = 0.0000%). An examination of the studentized residuals revealed that none of the studies had a value larger than ±2.6383 and hence there was no indication of outliers in the context of this model. According to the Cook’s distances, none of the studies could be considered to be overly influential. Neither the rank correlation nor the regression test indicated any funnel plot asymmetry (*p* = 1.0000 and *p* = 0.5069, respectively) [Supplementary Materials].

Oxygen saturation lower than 90%: six studies were included in the analysis. The observed log risk ratios ranged from −1.0986 to 0.0000, with the majority of estimates being negative (83%). The estimated average log risk ratio based on the random-effects model was $\hat{\mu }$ = −0.6515 (95% CI: −1.3713 to 0.0683). Therefore, the average outcome did not differ significantly from zero (z = −1.7740, *p* = 0.0761). According to the Q-test, there was no significant amount of heterogeneity in the true outcomes (Q(5) = 0.3718, *p* = 0.9961, tau^2^ = 0.0000, I^2^ = 0.0000%). One study (Rosenstock CV et al., 2012) had a relatively large weight compared to the rest of the studies (i.e., weight ≥ 3/k, so a weight at least 3 times as large as having equal weights across studies). An examination of the studentized residuals revealed that none of the studies had a value larger than ±2.6383 and hence there was no indication of outliers in the context of this model. According to the Cook’s distances, one study (Rosenstock CV et al., 2012) could be considered to be overly influential. Neither the rank correlation nor the regression test indicated any funnel plot asymmetry (*p* = 1.0000 and *p* = 0.8618, respectively) [Supplementary Materials].

### 3.11. Airway Anticipated to Be Difficult

Intubation time: three studies were included in the analysis. The observed standardized mean differences ranged from −5.2993 to −1.3429, with the majority of estimates being negative (100%). The estimated average standardized mean difference based on the random-effects model was $\hat{\mu }$ = −3.1237 (95% CI: −4.8086 to −1.4388). Therefore, the average outcome differed significantly from zero (z = −3.6337, *p* = 0.0003). According to the Q-test, the true outcomes appear to be heterogeneous (Q(2) = 24.4765, *p* < 0.0001, tau^2^ = 2.0020, I^2^ = 91.8289%). A 95% prediction interval for the true outcomes is described by the range −6.3686 to 0.1212. Hence, although the average outcome is estimated to be negative, in some studies the true outcome may in fact be positive. An examination of the studentized residuals revealed that none of the studies had a value larger than ±2.3940 and hence there was no indication of outliers in the context of this model. According to the Cook’s distances, none of the studies could be considered to be overly influential. Neither the rank correlation nor the regression test indicated any funnel plot asymmetry (*p* = 1.0000 and *p* = 0.7819, respectively) [Supplementary Materials].

Secondary outcomes: a pooled analysis could not be conducted for secondary outcomes due to the fact that for these outcomes the studies are smaller than 3.

### 3.12. Certainty of the Evidence Assessment

**Figure 9:**
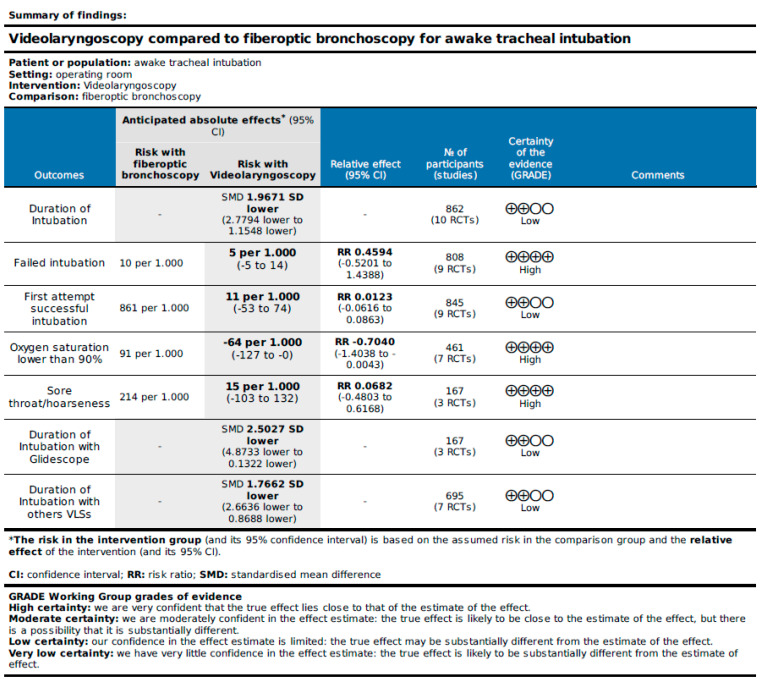
GRADE evidence profile for considered outcome.

The trials were evaluated overall using GRADE for RCTs, which analyzes the following elements for each outcome: number of studies, study design, risk of bias, inconsistency, indirectness, and imprecision. High-quality evidence was found for failed intubation, oxygen saturation lower than 90%, and sore throat/hoarseness; low-quality evidence was found for all other outcomes of interest (Figure 9).

### 3.13. Trial Sequential Analysis

**Figure 10:**
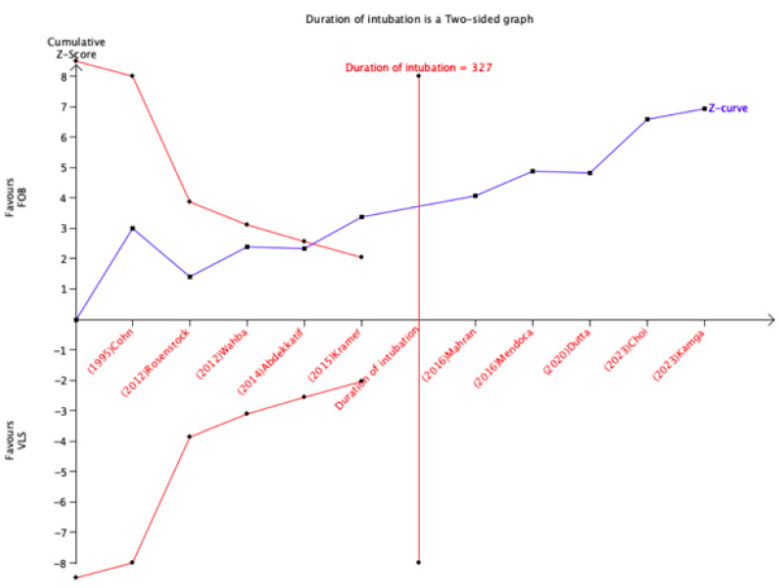
Trial sequential analysis for intubation time.

The TSA for the duration of intubation adjusted 95% CI ranged from −66.31 to −37.09. The cumulative Z curve crossed the conventional boundary for benefit, the sequential study futility boundary for benefit, and reached the size of the required information (327 to 314), suggesting that the current evidence was conclusive and that no further study of this outcome is needed (Figure 10). However, the cumulative Z-curve for failed intubation, first successful intubation attempt, oxygen saturation less than 90%, and sore throat/hoarseness did not cross either the conventional limit for benefit or the sequential study futility limit for benefit, suggesting that the current evidence for such outcomes was inconclusive and further studies are needed.

## 4. Discussion

This systematic review and meta-analysis aimed to assess the efficacy and safety of videolaryngoscopy compared to fiberoptic bronchoscopy for awake tracheal intubation. Pooled analysis revealed that videolaryngoscopy reduced the duration of intubation compared to fiberoptic bronchoscopy, a finding further supported by subgroup analysis based on the type of videolaryngoscope used. Moreover, the trial sequential analysis (TSA) confirmed the conclusiveness of this evidence.

The analysis revealed another intriguing finding: intubation time was significantly lower when intubation was achieved using a videolaryngoscope with sedation not targeted on the Ramsay level. This could be attributed to the absence of a predefined target sedation level, leading to higher doses of sedative and hypnotic drugs and consequently deeper sedation levels. This deeper sedation may facilitate the intubation procedure, resulting in a quicker process compared to when a target sedation level is established. Calculating the Ramsay score in studies without a predetermined sedation target could provide further insight into this observation highlighted by our analysis.

Additionally, two subgroup analyses revealed that videolaryngoscopy reduces intubation time in both anticipated airways, whether normal or difficult.

Furthermore, our analysis indicated that videolaryngoscopy slightly decreased the risk of experiencing oxygen saturation below 90% during the procedure. However, no statistically significant difference was observed between the two techniques regarding failed intubation, the first successful intubation attempt, and the occurrence of sore throat/hoarseness. Regarding patient-reported satisfaction, we were unable to conduct a pooled analysis due to the variability in evaluation methods used across the trials to assess this outcome.

Two prior meta-analyses have assessed the efficacy and safety of videolaryngoscopy versus fiberoptic bronchoscopy for awake intubation. Alhomary and colleagues conducted a meta-analysis of seven RCTs and reported that videolaryngoscopy was associated with a shorter intubation time compared to fiberoptic bronchoscopy. However, they found no significant difference between the two techniques in terms of failed intubation, first-attempt success rate, patient satisfaction, or intubation-related complications [31].

Jiang et al. conducted a meta-analysis of six RCTs and similarly found that intubation time was shorter when using videolaryngoscopy compared to fiberoptic bronchoscopy. They reported no differences between the two groups for other outcomes [32]. Like in their meta-analysis, as well as in ours, a high degree of heterogeneity among the studies was detected regarding intubation time. However, our meta-analysis not only confirmed the reduction in intubation time with the use of fiberoptic bronchoscopy but also, for the first time in the literature, provided a conclusive trial sequential analysis (TSA) on this topic. Remarkably, TSA on the comparison of awake intubation between fiberoptic bronchoscopy and videolaryngoscopy had not been previously performed. Our TSA supported the notion that further studies could enhance the assessment of this considered outcome.

Therefore, our meta-analysis revealed that the utilization of videolaryngoscopy decreases the risk of experiencing oxygen saturation below 90% during the intubation maneuver. Importantly, no significant heterogeneity was detected for this outcome. This novel finding can be attributed to the inclusion of a larger number of trials and patients, more than doubling compared to previous meta-analyses. However, despite this, the trial sequential analysis (TSA) indicated that the current evidence was inconclusive for this outcome.

The high degree of heterogeneity observed among the studies included in the analysis can be attributed to several factors. Firstly, not all studies enrolled participants with known or anticipated difficult airways, and even among those that did, the criteria used for assessment varied significantly between studies. Specifically, studies conducted by Choi et al., Cohn et al., Dutta et al., Kamga et al., and Wahba et al. did not require known or anticipated difficult airways as inclusion criteria. Additionally, it is worth noting that all these studies, except for Kamga et al., included patients with cervical spine disease or traumatic cervical spine injuries, necessitating the maintenance of a neutral cervical spine position during the intubation procedure [21,22,23,24,30]. On the other hand, the remaining studies included in the meta-analysis recruited patients with known or anticipated difficult airways; however, airway assessment was not standardized across studies [20,25,26,27,28,29]. Secondly, the studies in our meta-analysis encompassed patients in various clinical contexts, such as obese patients undergoing bariatric surgery [20,28], patients with cervical spinal pathology [21,22,23,24,30], patients undergoing maxillofacial surgery [25,26,29], and patients undergoing gynecological, urological, and abdominal surgery [29]. Moreover, the studies included in the meta-analysis defined the operator’s experience in intubation with the videolaryngoscope and with fiberoptic bronchoscopy using different criteria; consequently, the intubations in our analysis were conducted by operators with varying levels of experience in using the two devices. Furthermore, premedication, sedation, and local anesthesia protocols for awake intubation differed among the studies included in the meta-analysis. Finally, the measurement of intubation time was different among the studies analyzed; in fact, five studies used the mean and standard deviation, while the other five used the median and interquartile range to assess this outcome.

It is crucial to acknowledge several limitations in our meta-analysis. Firstly, as discussed previously, we conducted a meta-analysis on an outcome with a high degree of heterogeneity, and therefore, the results should be interpreted cautiously despite the utilization of the random-effects model. Secondly, the variability in the types of videolaryngoscopes used in different studies prevented us from performing sufficient subgroup analyses. The Glidescope was the only videolaryngoscope used in more than two studies. Thirdly, although our assessment demonstrated a low risk of bias in most domains among the majority of included studies, blinding of operators and outcome assessors was not feasible, which may raise concerns regarding our results. Fourthly, due to the variability in evaluation methods used across the various trials to assess patient satisfaction, we were unable to conduct a pooled analysis of this data. Fifthly, the definition of an operator as “expert” in performing intubation with both devices varied among the studies analyzed. Sixthly, the included studies employed non-uniform sedation protocols and target sedation levels. Lastly, all included studies performed elective intubation maneuvers on surgical patients; therefore, it is possible that these findings could differ in an emergency context.

## 5. Conclusions

In this systematic review and meta-analysis comparing videolaryngoscopy to fiberoptic bronchoscopy for awake tracheal intubation, we observed a reduction in the duration of intubation time and a decreased risk of oxygen saturation falling below 90% during the procedure when videolaryngoscopy was utilized. However, it is important to note that regarding intubation time, the results should be interpreted with caution due to the high degree of heterogeneity detected among the analyzed studies, which represents a significant limitation.

## Figures and Tables

**Table 1:** Baseline characteristics of studies included in the systematic review and meta-analysis. ASA: American Society of Anesthesiologists physical status classification; BMI: Body mass index; EGRI: El-Ganzouri Risk Index; ENT: Ear, Nose, and Throat; FOB: Fibreoptic bronchoscopy; SARI: Simplified Airway Risk Index; VL: Videolaryngoscopy.

Study	Type of VLS Device	Sample Size	Type of Surgery	Inclusion Criteria	Exclusion Criteria	Experience of Operator	Sedation	Intubation
Abdellatif AA et al., 2014 [20]	Glidescope	VL: 31 FOB: 32	Laparoscopic bariatric	BMI ≥ 40, EGRI ≥ 4	Age < 18 or >60, ASA class ≥ 4, severe mental illness, mouth opening < 15 mm, poor dentition, controindications to drugs used during procedure, patient refusal	Two anaesthetists with experience of more than 100 successful tracheal intubations using both devices	Premedication with glycopyrrolate; topical anaesthesia with lidocaine; remifentanil TCI (target Ramsay score of 3)	Oral
Choi S et al., 2023 [21]	AceScope	VL: 166 FOB: 164	Elective cervical spine surgery	Patients aged 20–80 years scheduled for elective cervical spine surgery under general anaesthesia	Upper airway lesion (tumour; polyp; trauma; abscess; inflammation; or foreign body), history of upper airway surgery or radiotherapy, high risk of pulmonary aspiration, coagulopathy, or ASA class > 3	Five consultant anaesthetists with a collective history of more than 30 successful tracheal intubations using both devices	Propofol TCI	Oral
Cohn AI et al., 1995 [22]	Bullard	VL: 8 FOB: 9	Cervical spine	Adults, ASA class I–III, scheduled for neurosurgical correction of a cervical spine problem	Not declared	Senior trainees or consultant anaesthetists with experience in at least 10 successful tracheal intubations using both devices	Fentanyl, midazolam, and droperidol; topical anaesthesia with lidocaine	Oral
Dutta K et al., 2020 [23]	McGrath	VL: 23 FOB: 23	Elective cervical spine surgery	Patients aged 18–65 years and ASA class I–II.	Oropharyngeal pathology, mouth opening < 2.5 cm, emergency surgery, pregnancy, or refusal of consent	One anesthesiologist who has adequate experience in performing intubations with both these techniques (>25 intubations)	Premedication with glycopyrrolate; fentanyl; lidocaine for bilateral superior laryngeal block and trans-tracheal administration; the oropharynx was anesthetized with lidocaine spray	Oral
Kamga H et al., 2023 [24]	Airtraq	VL: 39 FOB: 39	Elective	Age ≥ 18, required awake tracheal intubation	Mouth opening < 16 mm, surgery involved the mouth or nose	Expert consultant anaesthetists with experience in at least 10 flexible bronchoscopy awake tracheal intubations without supervision or trainees under the supervision of an expert consultant	Remifentanil TCI (Ramsay score of 2); local anaesthesia with licocaine of the upper airway was performed with a combination of topical airway anaesthesia, superior laryngeal nerve blockade, and a tracheal block	Oral
Kramer A et al., 2015 [25]	C-MAC D-BLADE	VL: 50 FOB: 50	Oral and maxillofacial	Age > 18, mouth opening > 13 mm, with at least one criterion for an anticipated difficult intubation (a modified Mallampati score of 4; an inter-incisor distance < 2.5 cm; a documented history of a difficult intubation; or an obstacle for a standard intubation like tumour or swelling).	Dental abscesses, ASA class ≥ 4	Nine anaesthetists with at least 1 year of experience in oral and maxillofacial anaesthesia and experience of successful tracheal intubations using C-MAC and FOB > 20 and 50 times, respectively	Midazolam and remifentanil; topical anaesthesia with lidocaine	Nasal
Mahran EA et al., 2016 [26]	Glidescope	VL: 27 FOB: 27	Oropharyngeal cancer	Age ≥ 20 or ≤60, ASA class I–II, Mallampati Score II–III	Patient refusal, restricted mouth opening, bleeding tendency, or any contraindication to nasal intubation	Two anaesthetists experienced with both devices	Premedication with glycopyrrolate; phenylephrine nasal drops; remifentanil infusion (Ramsay score of 3); topical anaesthesia with lidocaine	Nasal
Mendonca C et al., 2016 [27]	Pentax AWS	VL: 20 FOB: 20	Elective	Adults, high Mallampati score, limited neck extension, limited jaw protrusion, requirement to maintain the cervical spine in the neutral position during intubation	Required nasal intubation, age < 18, pregnancy, mouth opening < 25 mm	Consultant anaesthetist experienced in head and neck surgery and with experience of successful tracheal intubations using Pentax AWS and FOB > 30 and 100 times, respectively	Premedication with glycopyrrolate; midazolam and remifentanil infusion (Ramsay score of 2–3); topical anaesthesia with lidocaine	Oral
Moore A et al., 2017 [28]	Glidescope	VL: 5 FOB: 6	Bariatric	SARI ≥ 4	Not declared	Four anaesthetists with experience of more than 40 successful tracheal intubations using both devices	Premedication with glycopyrrolate; midazolam and remifentanil infusion; topical anaesthesia with lidocaine	Oral
Rosenstock CV et al., 2012 [29]	McGrath	VL: 41 FOB: 43	Gynecologic, abdominal, urologic, ENT	Adults, ASA class I–III, anticipated difficult laryngoscopy or intubation, SARI ≥ 4	Age ≤ 18, ASA class IV–V, mouth opening < 15 mm, poor dentaition, surgeon request of nasal intubation, contraindication for transtracheal injection	Six anaesthetists experienced in difficult airway management and both airway devices	Premedication with glycopyrrolate; remifentanil infusion and a bolus of remifentanil or propofol (Ramsay score of 2–4); topical anaesthesia with lidocaine; trans-tracheal injection	Oral
Wahba SSTT et al., 2012 [30]	Glidescope	VL: 25 FOB: 25	Cervical spine	Adults, ASA class I–III	BMI ≥ 35, obstructive airway disease, cardiovascular disease, apparent difficult airway, patient refusal	Consultant anaesthetist with experience of more than 100 successful tracheal intubations using both devices	Premedication with glycopyrrolate; midazolam and remifentanil infusion; topical anaesthesia with lidocaine	Oral

**Table 2:** Risk of bias assessed by using the Cochrane Risk of Bias Tool for RCTs.

Study ID	D1	D2	D3	D4	D5	Overall
Abdellatif AA et al., 2014 [20]	-	+	+	+	-	-
Choi S et al., 2023 [21]	+	+	+	+	+	+
Dutta K et al., 2020 [23]	+	+	+	+	+	+
Kamga H et al., 2023 [24]	+	+	+	+	+	+
Kramer A et al., 2015 [25]	+	+	+	+	-	+
Mahran EA et al., 2016 [26]	+	+	+	+	-	+
Mendonca C et al., 2016 [27]	-	+	+	+	+	+
Rosenstock CV et al., 2012 [29]	+	+	+	+	-	+
Wahba SSTT et al., 2012 [30]	-	-	+	+	-	-

Domains: D1: Bias due to randomization. D2: Bias due to deviations from intended intervention. D3: Bias due to missing data. D4: Bias due to outcome measurement. D5: Bias due to selection of reported result. Judgement: -: Some concern. +: Low.

## Data Availability

The datasets used and/or analyzed during the current study are available from the corresponding author on reasonable request.

## Supplementary Materials

The following supporting information can be downloaded at: https://www.mdpi.com/article/10.3390/jcm13113186/s1, Figure S1: Forest plot for the comparison of first attempt successful intubation for studies with targeted sedation on Ramsay score. Figure S2: Publication bias assessment of studies with targeted sedation on Ramsay score. Figure S3: Forest plot for the comparison of first attempt successful intubation for studies without targeted sedation on Ramsay score. Figure S4: Publication bias assessment of studies without targeted sedation on Ramsay score. Figure S5: Forest plot for the comparison of failed intubation for studies with targeted sedation on Ramsay score. Figure S6: Publication bias assessment of studies with targeted sedation on Ramsay score. Figure S7: Forest plot for the comparison of failed intubation for studies without targeted sedation on Ramsay score. Figure S8: Publication bias assessment of studies without targeted sedation on Ramsay score. Figure S9: Forest plot for the comparison of oxygen saturation lower than 90% for studies with targeted sedation on Ramsay score. Figure S10: Publication bias assessment of studies with targeted sedation on Ramsay score. Figure S11: Forest plot for the comparison of oxygen saturation lower than 90% for studies without targeted sedation on Ramsay score. Figure S12: Publication bias assessment of studies without targeted sedation on Ramsay score. Figure S13: Forest plot for the comparison of intubation time for studies with targeted sedation on Ramsay score. Figure S14: Publication bias assessment of studies with targeted sedation on Ramsay score. Figure S15: Forest plot for the comparison of intubation time for studies without targeted sedation on Ramsay score. Figure S16: Publication bias assessment of studies without targeted sedation on Ramsay score. Figure S17: Forest plot for the comparison of intubation time in airway anticipated to be normal. Figure S18: Forest plot for the comparison of first attempt successful intubation in airway anticipated to be normal. Figure S19: Forest plot for the comparison of failed intubation in airway anticipated to be normal. Figure S20: Forest plot for the comparison of oxygen saturation lower than 90% in airway anticipated to be normal. Figure S21: Forest plot for the comparison of intubation time in airway anticipated to be difficult.
