# Supplementary material for: Videolaryngoscopy versus Fiberoptic Bronchoscopy for Awake Tracheal Intubation: A Systematic Review and Meta-Analysis of Randomized Controlled Trials
Source: J Clin Med. 2024 May 29;13(11):3186. doi: 10.3390/jcm13113186 (PMC11173084; doi:10.3390/jcm13113186)
Supplement: Supplementary file 1 [file jcm-13-03186-s001.zip › jcm-2983057-supplementary.pdf]

Supplementary Materials

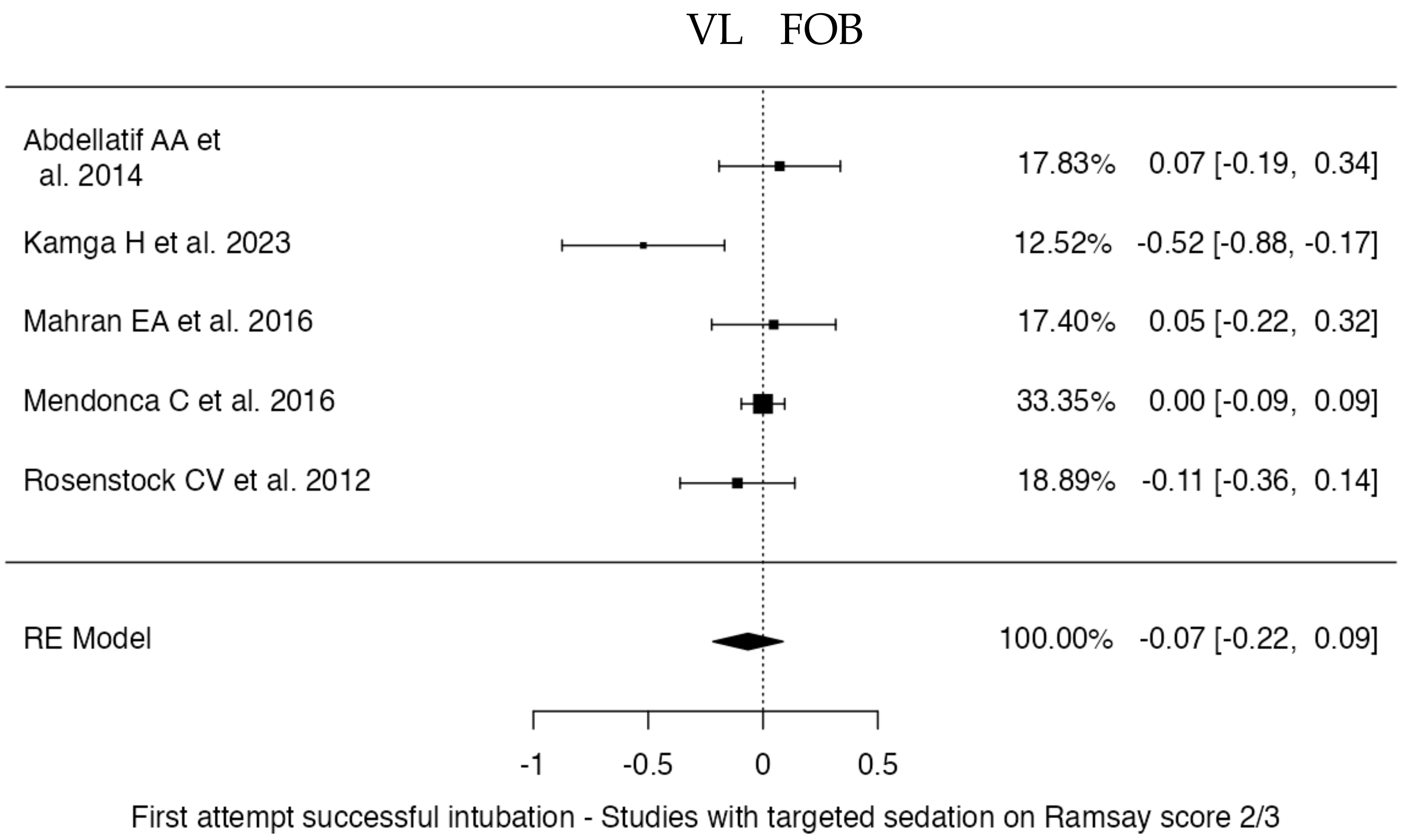

Figure S1: Forest plot for the comparison of first attempt successful intubation for studies with targeted sedation on Ramsay score.

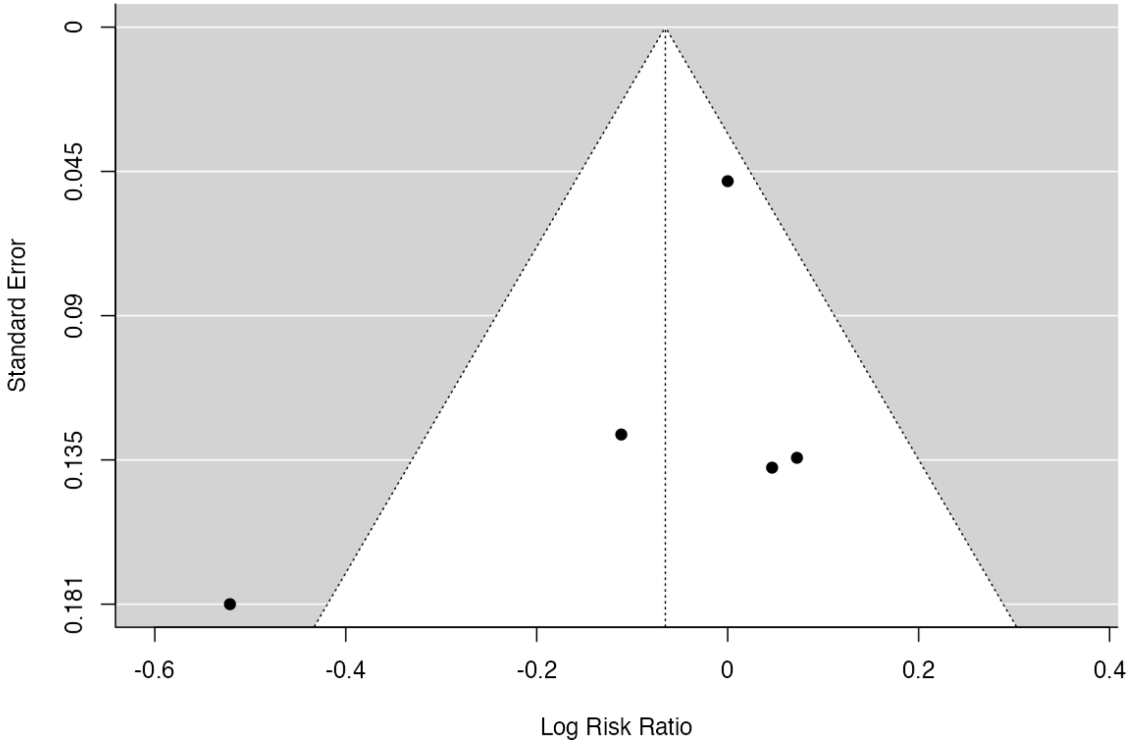

Publication Bias Assessment

Fail-Safe N Analysis (File Drawer Analysis)

Fail-safe N	p
0.000	0.098

Nota. Fail-safe N Calculation Using the Rosenthal Approach

Rank Correlation Test for Funnel Plot Asymmetry

Kendall's Tau	p
–0.600	0.233

Regression Test for Funnel Plot Asymmetry

Z	p
–1.116	0.265

Figure S2: Publication bias assessment of studies with targeted sedation on Ramsay score.

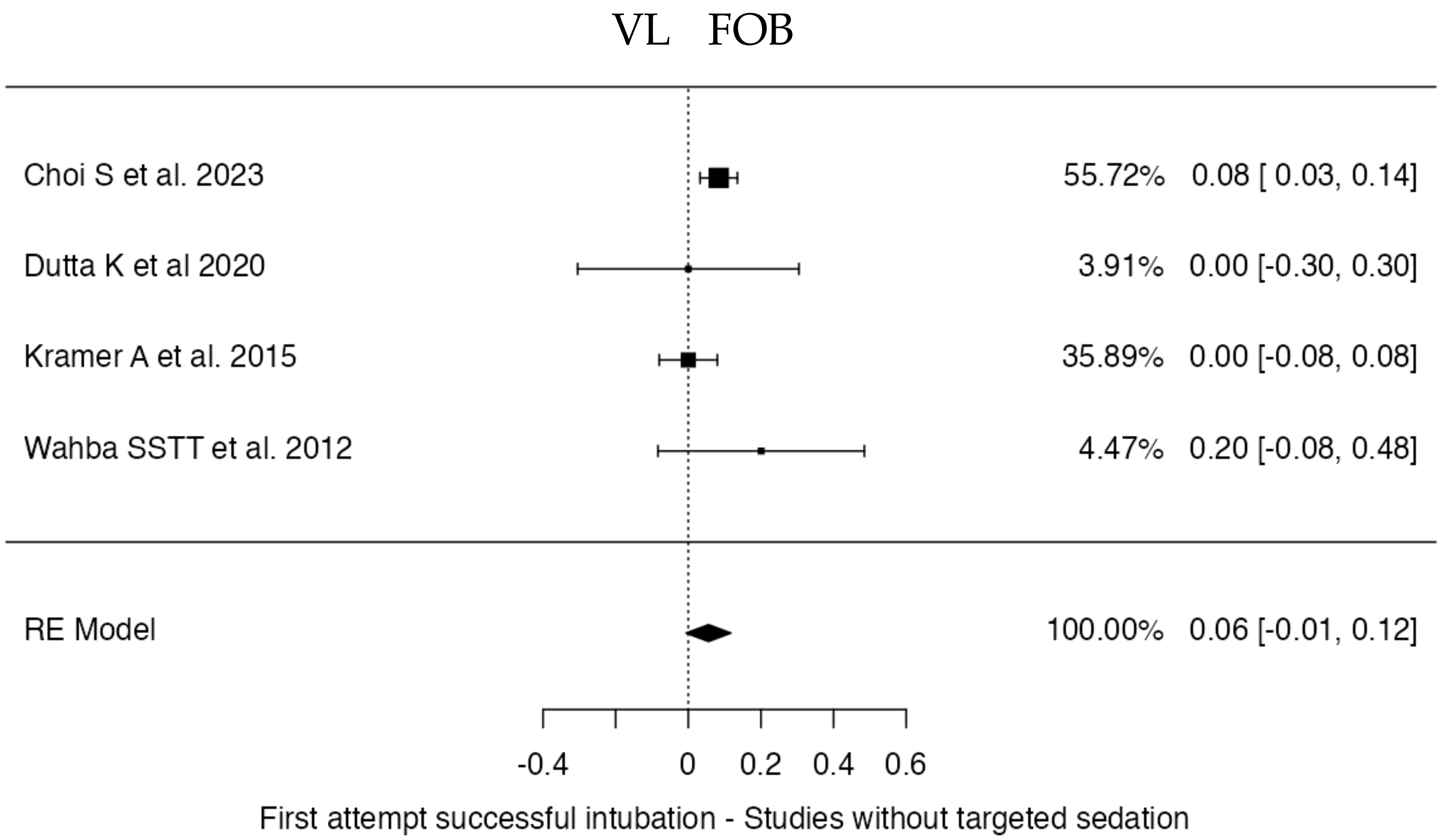

Figure S3: Forest plot for the comparison of first attempt successful intubation for studies without targeted sedation on Ramsay score.

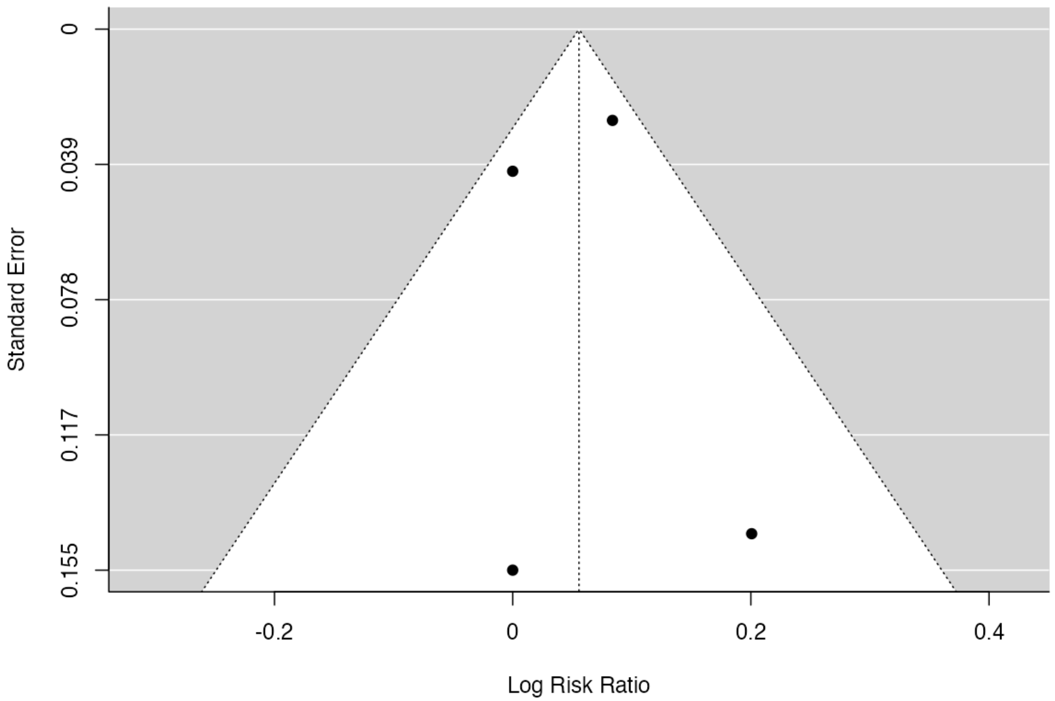

Publication Bias Assessment

Fail-Safe N Analysis (File Drawer Analysis)

Fail-safe N	p
4.000	0.011

Nota. Fail-safe N Calculation Using the Rosenthal Approach

Rank Correlation Test for Funnel Plot Asymmetry

Kendall's Tau	p
–0.333	0.750

Regression Test for Funnel Plot Asymmetry

Z	p
0.280	0.779

Figure S4: Publication bias assessment of studies without targeted sedation on Ramsay score.

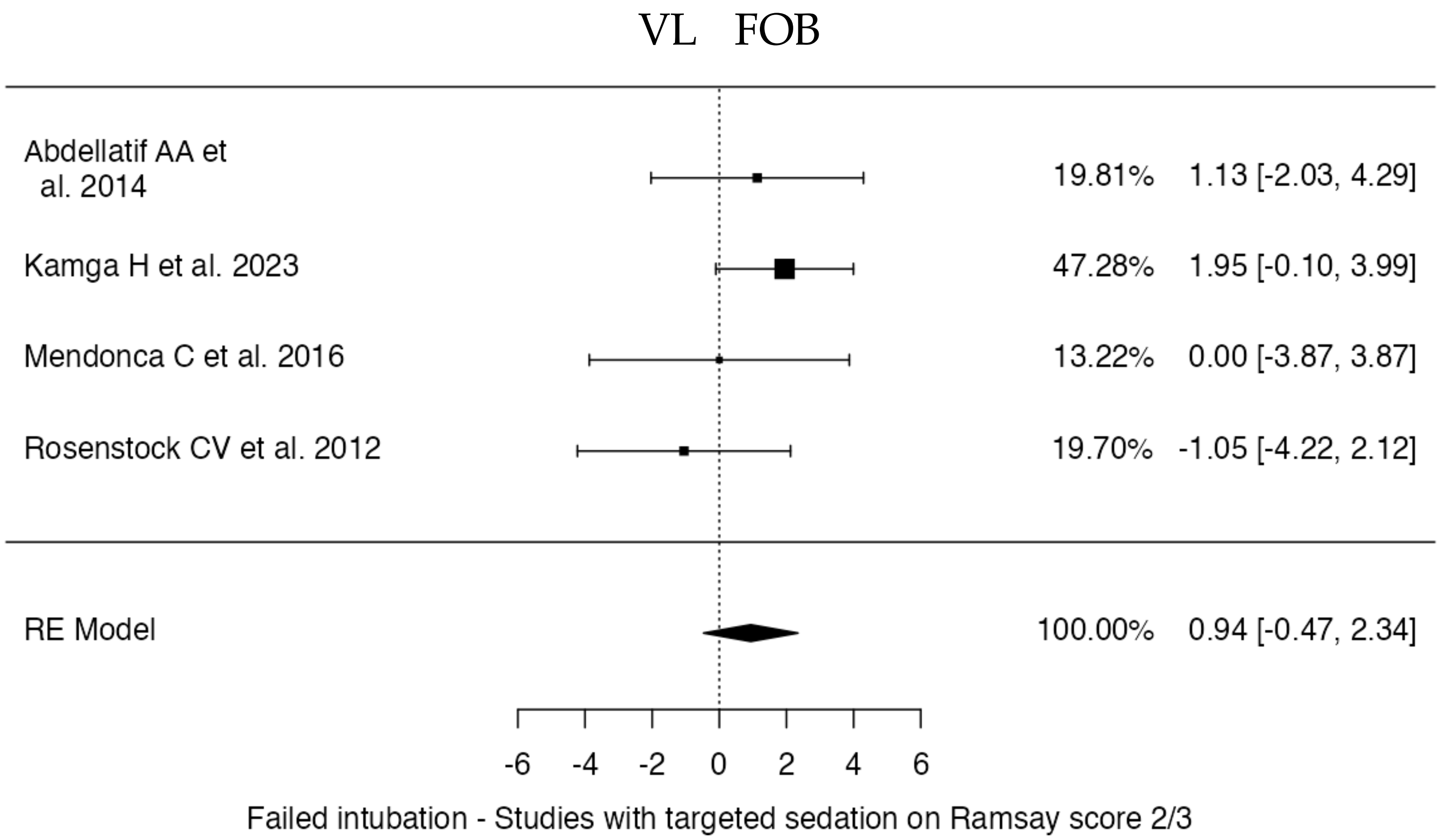

Figure S5: Forest plot for the comparison of failed intubation for studies with targeted sedation on Ramsay score.

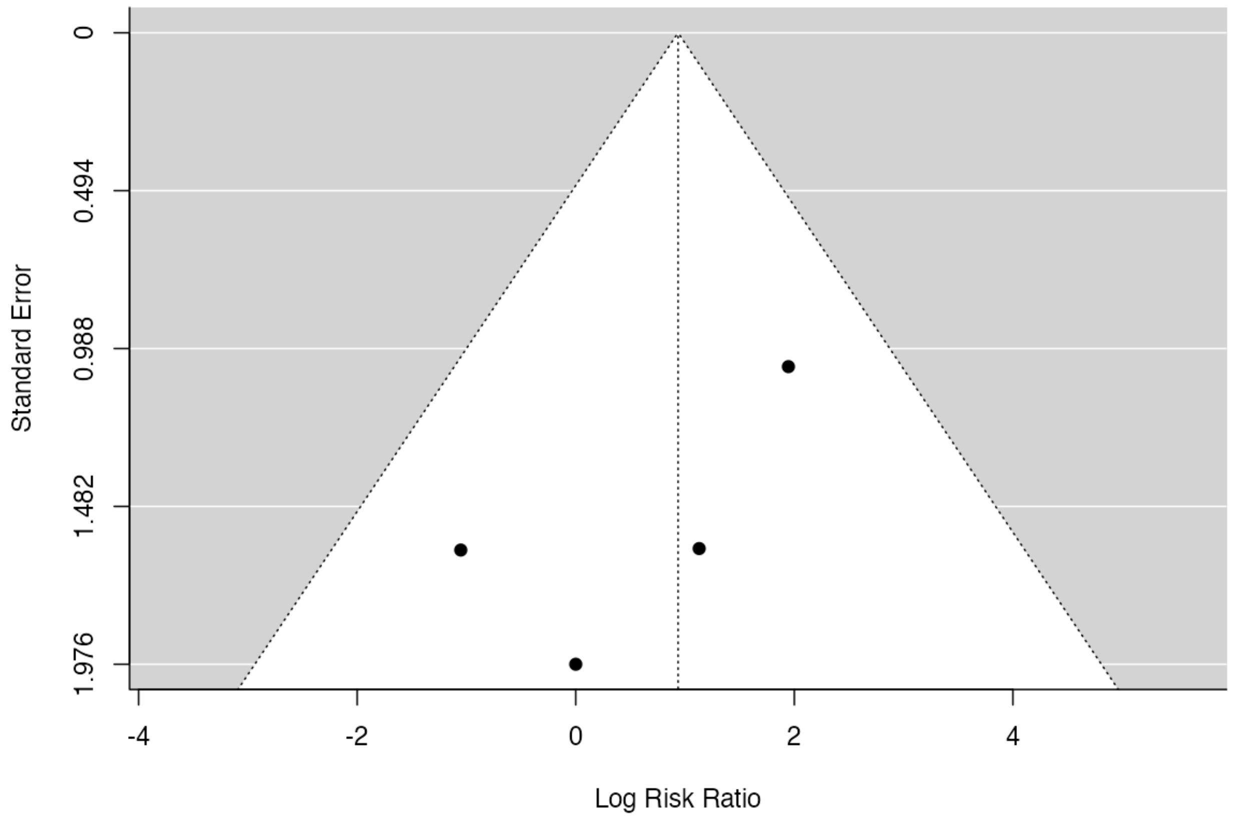

Fail-Safe N Analysis (File Drawer Analysis)

Fail-safe N	p
0.000	0.170

Nota. Fail-safe N Calculation Using the Rosenthal Approach

Rank Correlation Test for Funnel Plot Asymmetry

Kendall's Tau	p
–0.667	0.333

Regression Test for Funnel Plot Asymmetry

Z	p
–1.269	0.205

Figure S6: Publication bias assessment of studies with targeted sedation on Ramsay score.

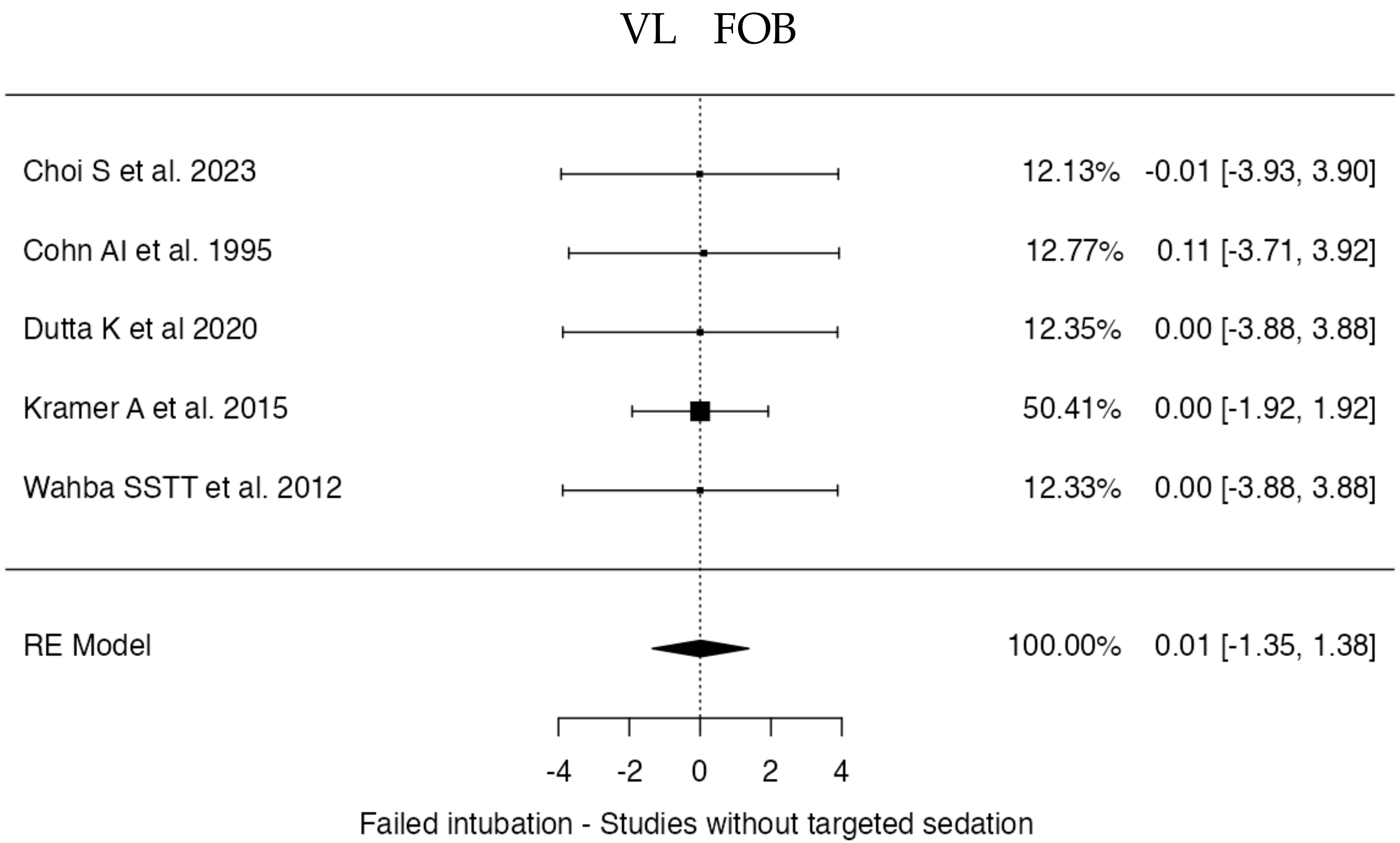

Figure S7: Forest plot for the comparison of failed intubation for studies without targeted sedation on Ramsay score.

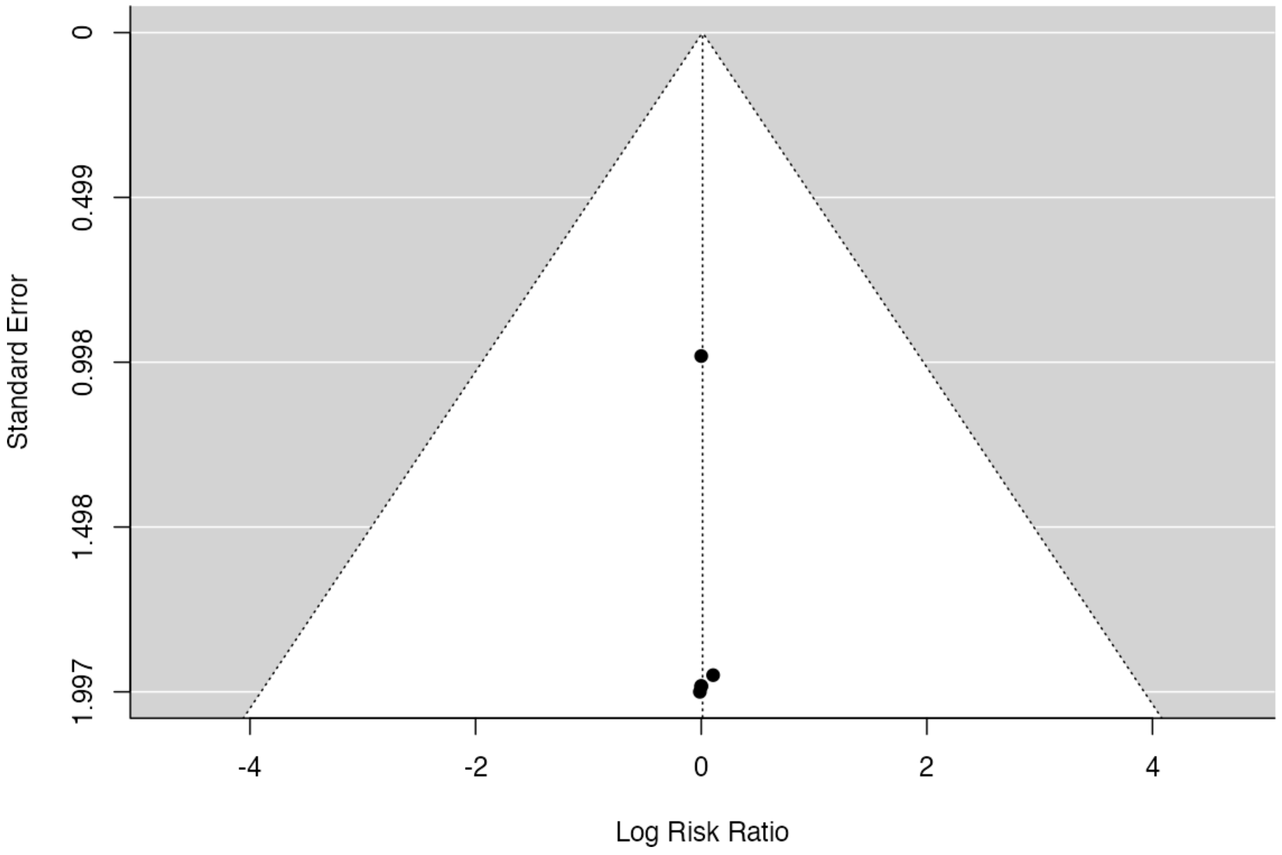

Publication Bias Assessment

Fail-Safe N Analysis (File Drawer Analysis)

Fail-safe N	p
0.000	0.491

Nota. Fail-safe N Calculation Using the Rosenthal Approach

Rank Correlation Test for Funnel Plot Asymmetry

Kendall's Tau	p
0.000	1.000

Regression Test for Funnel Plot Asymmetry

Z	p
0.016	0.987

Figure S8: Publication bias assessment of studies without targeted sedation on Ramsay score.

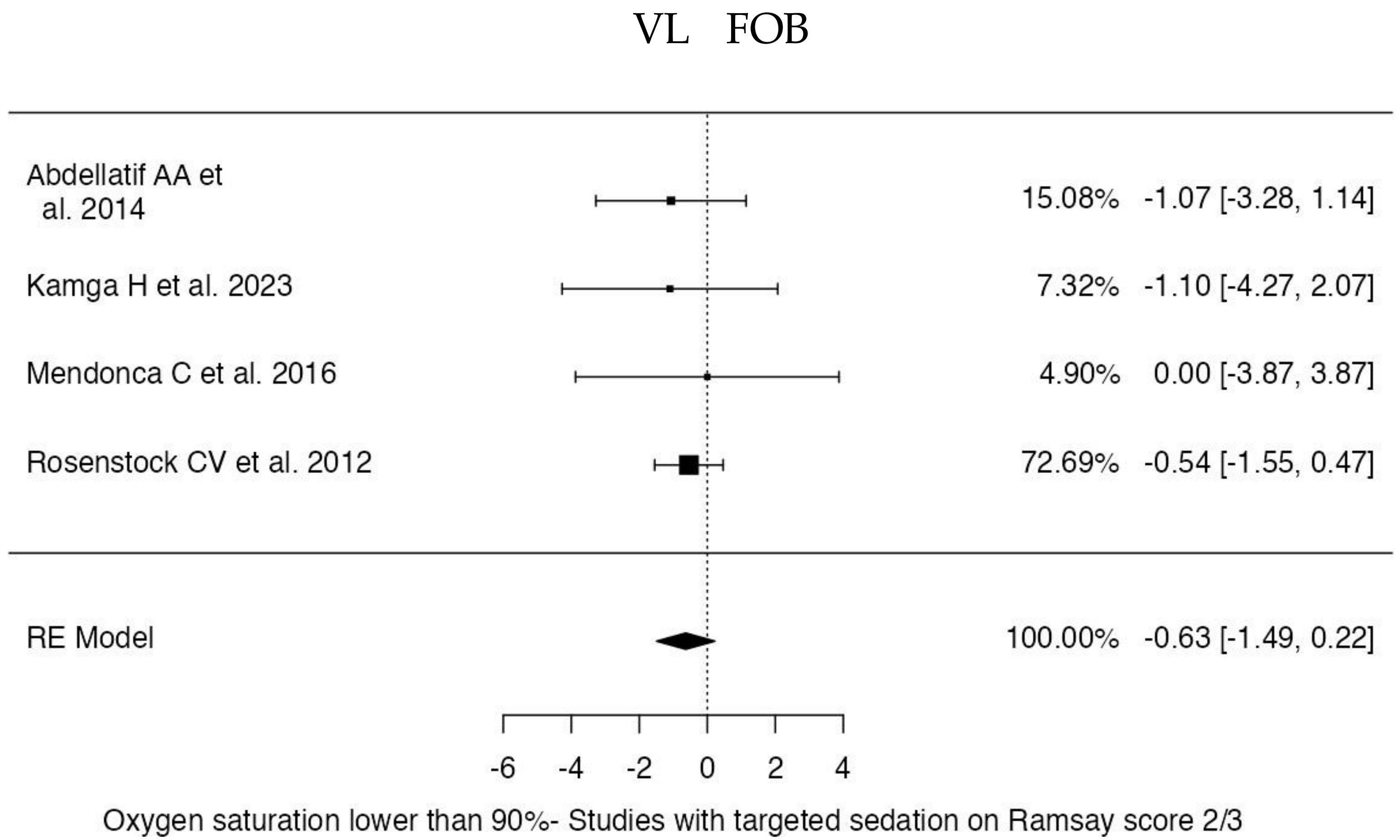

Figure S9: Forest plot for the comparison of oxygen saturation lower than 90% for studies with targeted sedation on Ramsay score.

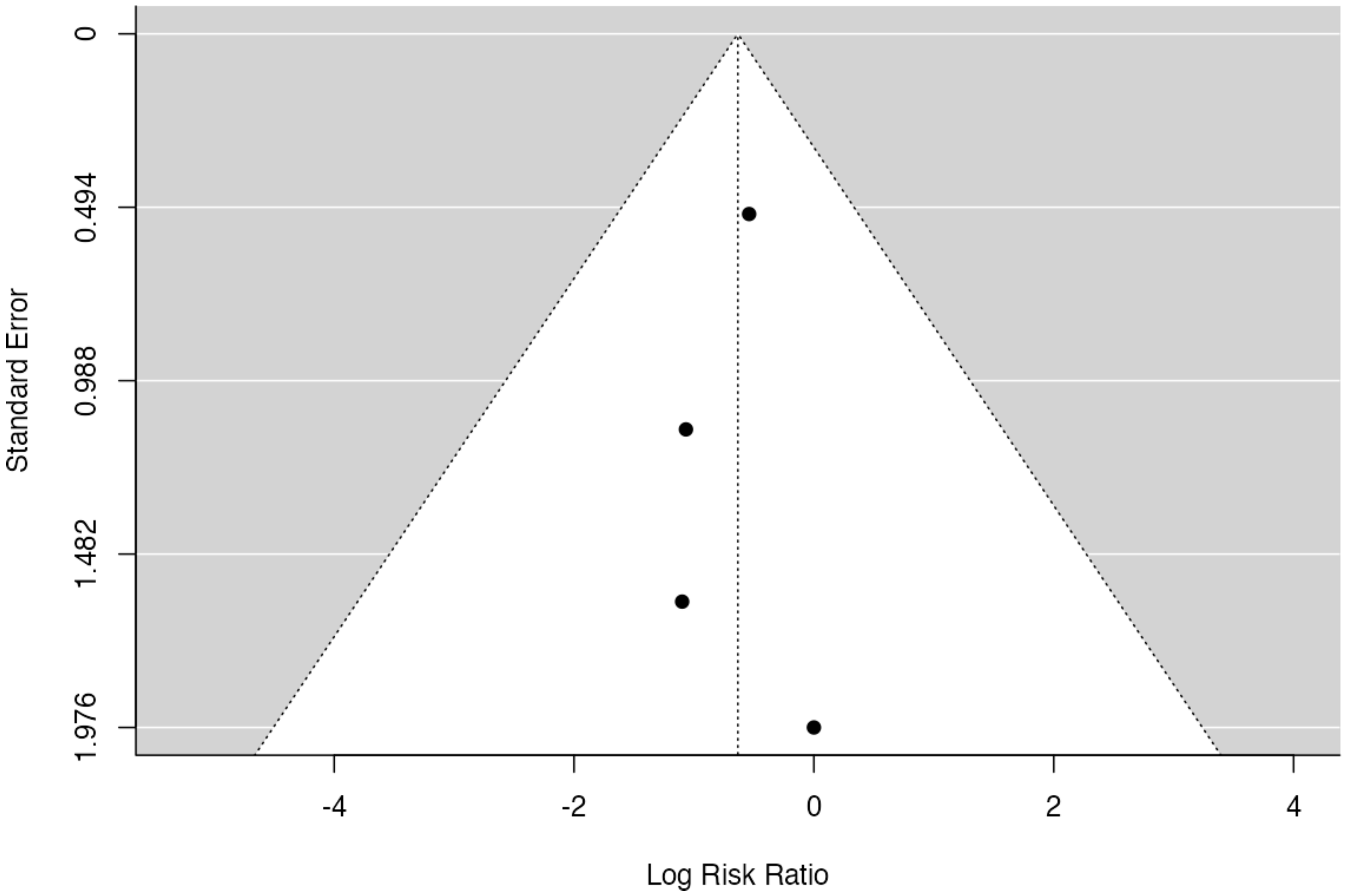

Fail-Safe N Analysis (File Drawer Analysis)

Fail-safe N	p
0.000	0.090

Nota. Fail-safe N Calculation Using the Rosenthal Approach

Rank Correlation Test for Funnel Plot Asymmetry

Kendall's Tau	p
0.000	1.000

Regression Test for Funnel Plot Asymmetry

Z	p
–0.168	0.867

Figure S10: Publication bias assessment of studies with targeted sedation on Ramsay score.

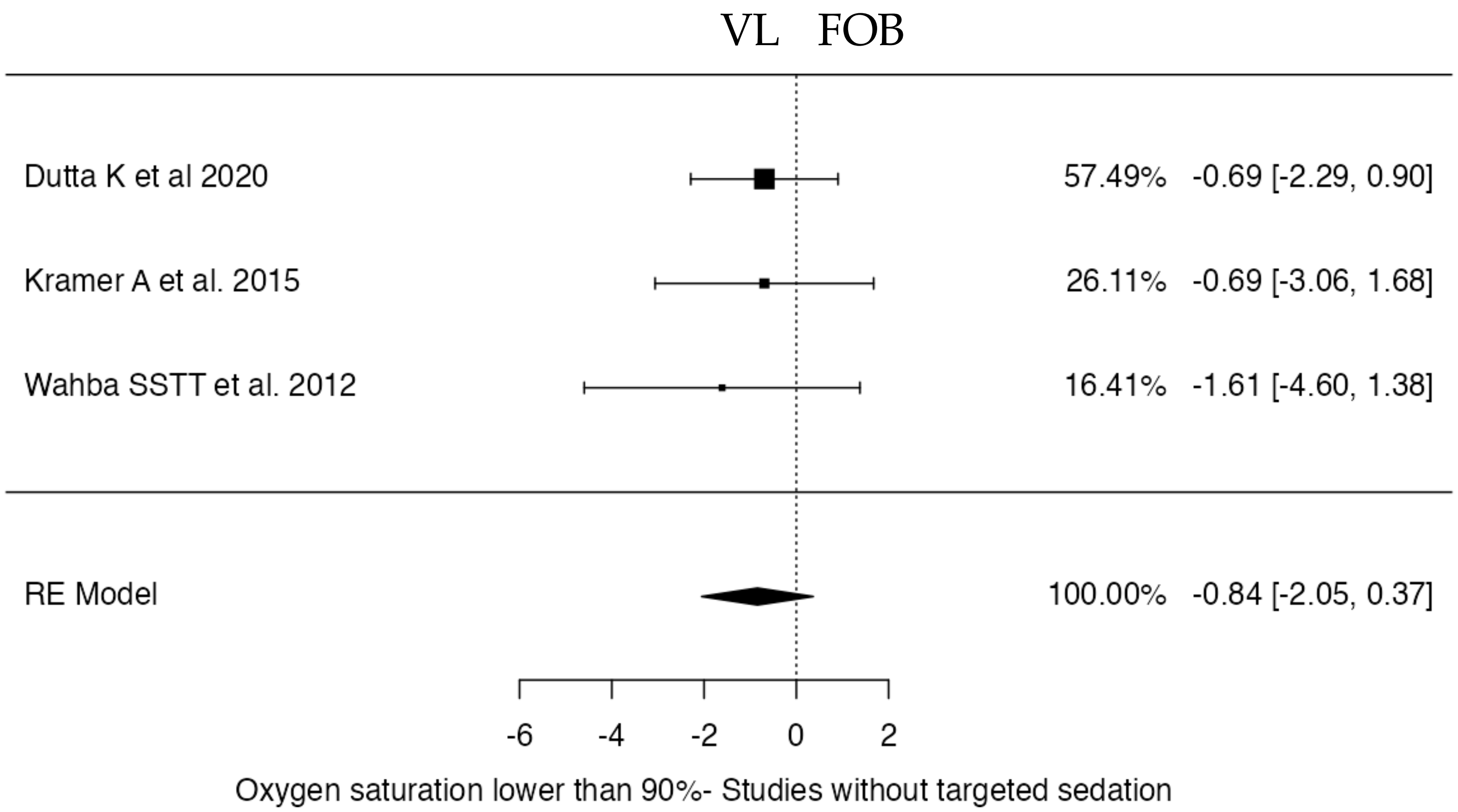

Figure S11: Forest plot for the comparison of oxygen saturation lower than 90% for studies without targeted sedation on Ramsay score.

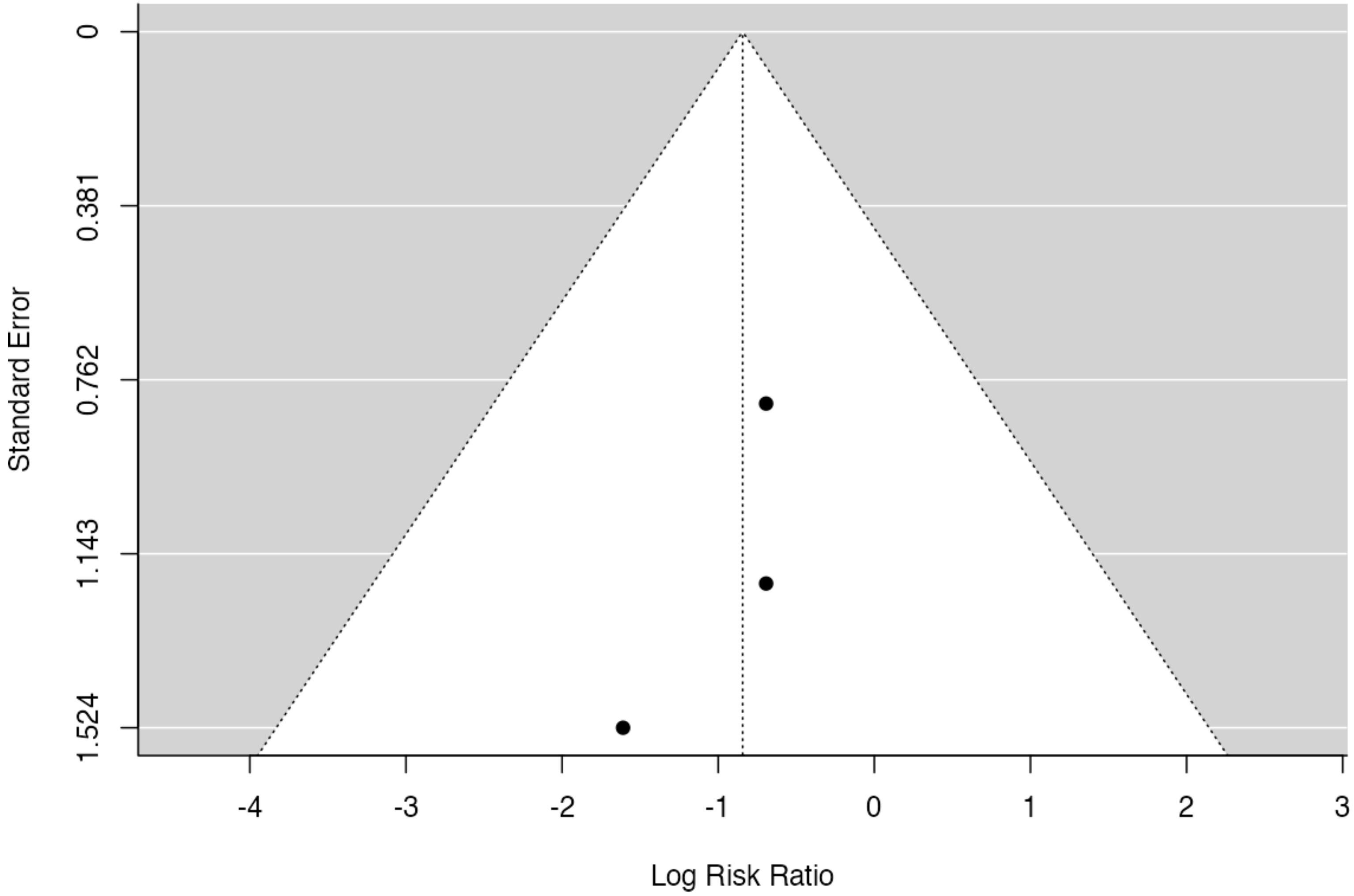

Fail-Safe N Analysis (File Drawer Analysis)

Fail-safe N	p
0.000	0.076

Nota. Fail-safe N Calculation Using the Rosenthal Approach

Rank Correlation Test for Funnel Plot Asymmetry

Kendall's Tau	p
−1.000	0.333

Regression Test for Funnel Plot Asymmetry

Z	p
−0.436	0.663

Figure S12: Publication bias assessment of studies without targeted sedation on Ramsay score.

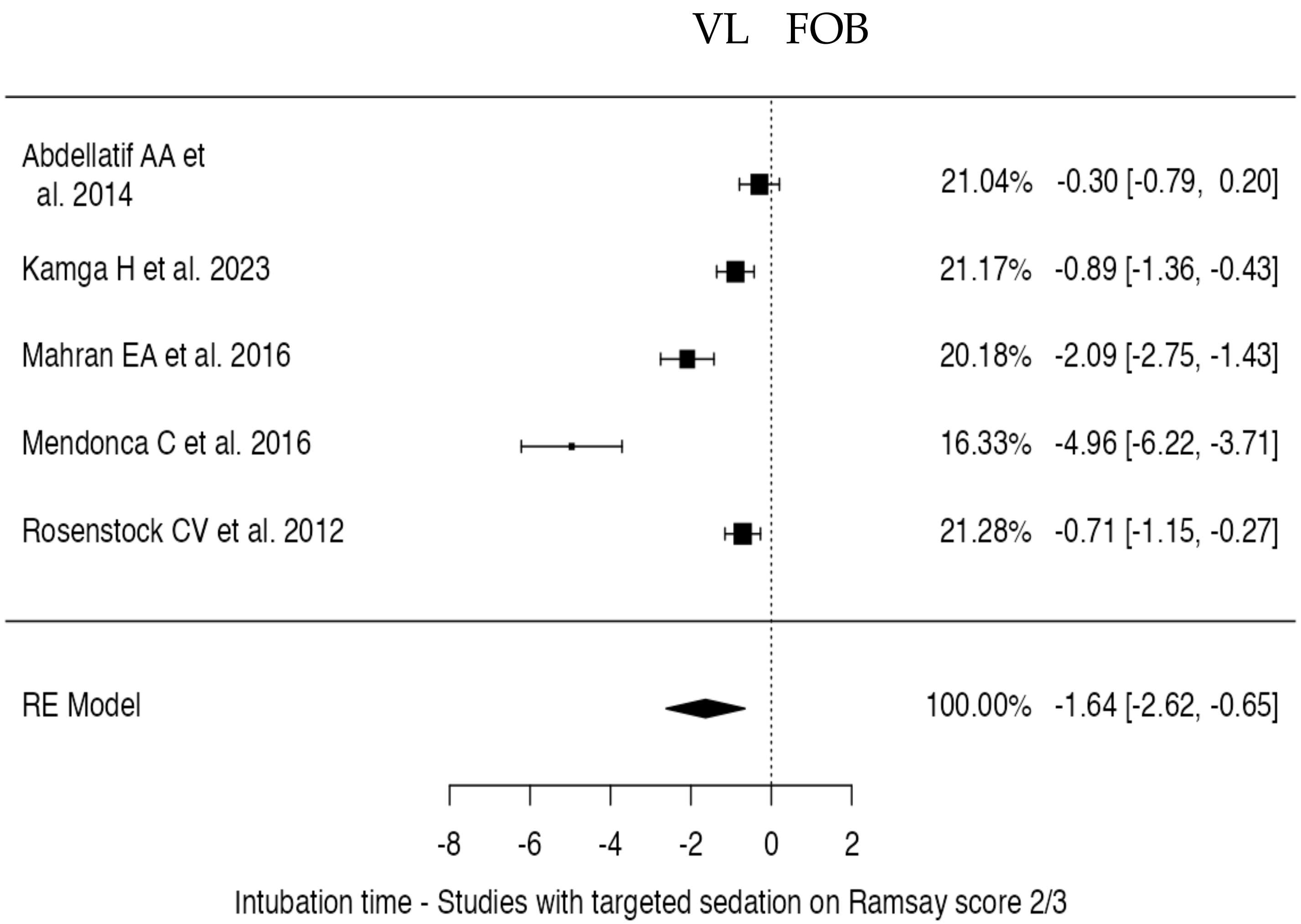

Figure S13: Forest plot for the comparison of intubation time for studies with targeted sedation on Ramsay score.

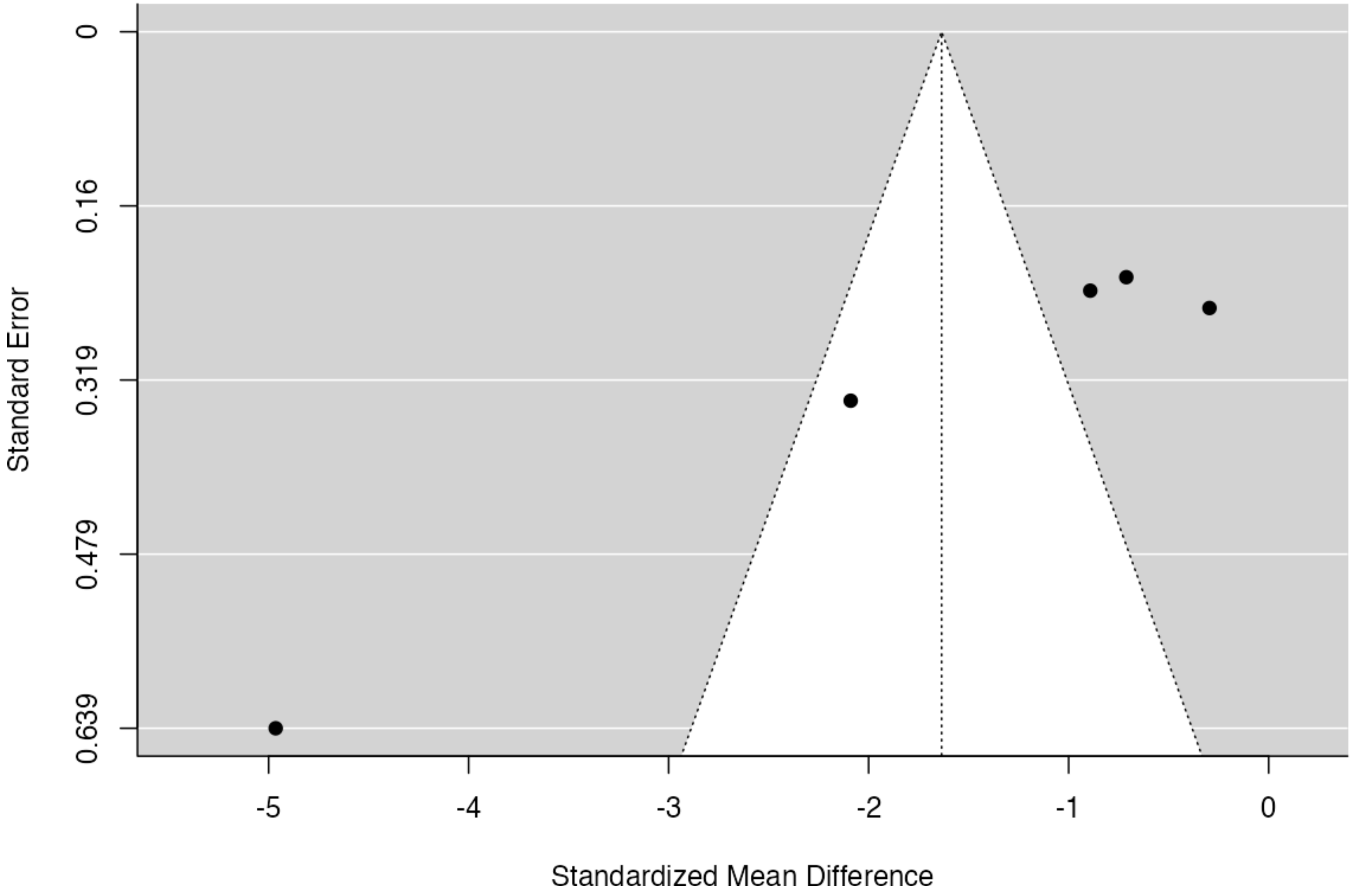

Publication Bias Assessment

Test Name	value	p
Fail-Safe N	175.000	<.001
Begg and Mazumdar Rank Correlation	–0.600	0.233
Egger's Regression	–6.048	<.001
Trim and Fill Number of Studies	0.000	.

Nota. Fail-safe N Calculation Using the Rosenthal Approach

Figure S14: Publication bias assessment of studies with targeted sedation on Ramsay score.

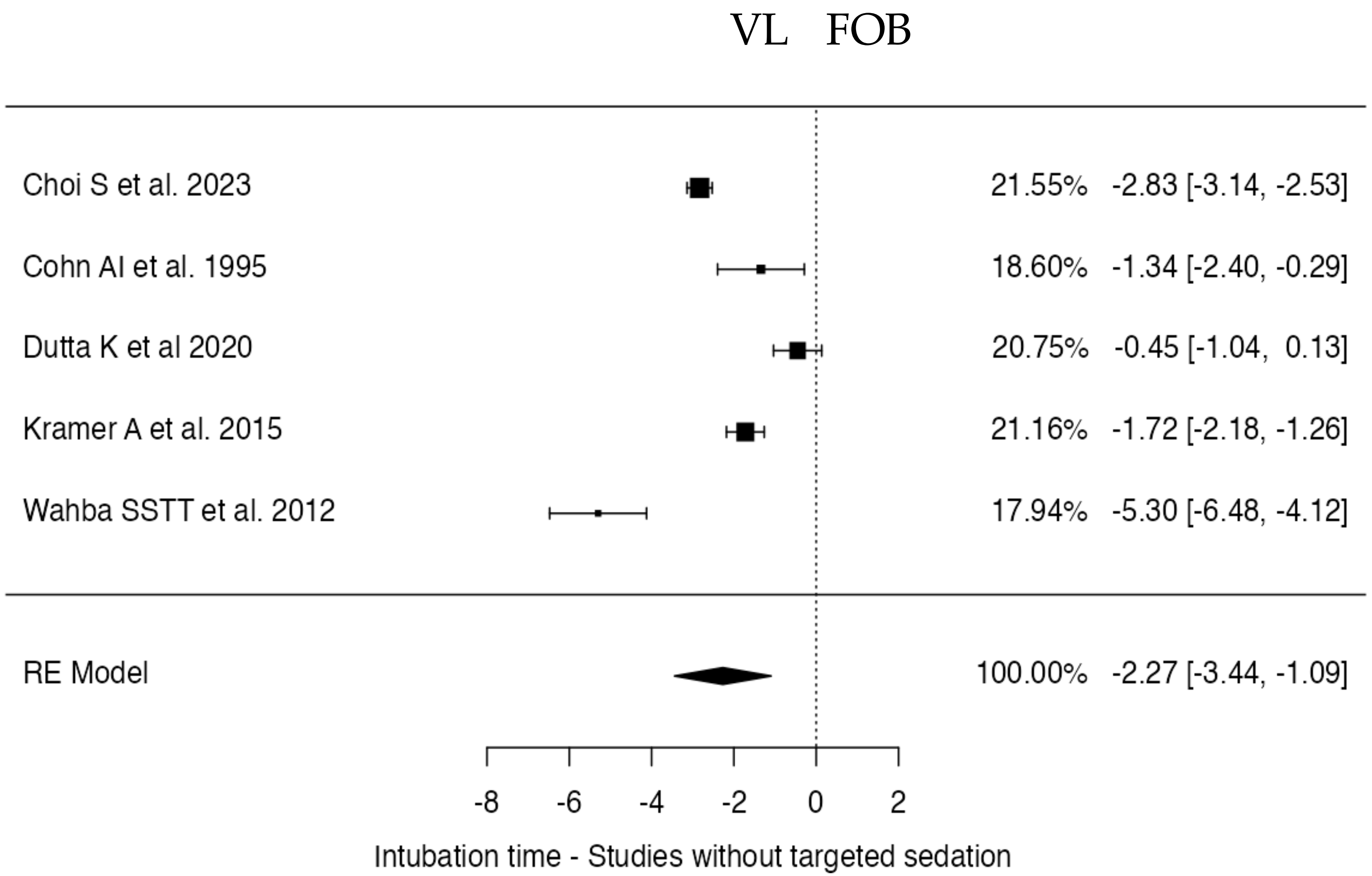

Figure S15: Forest plot for the comparison of intubation time for studies without targeted sedation on Ramsay score.

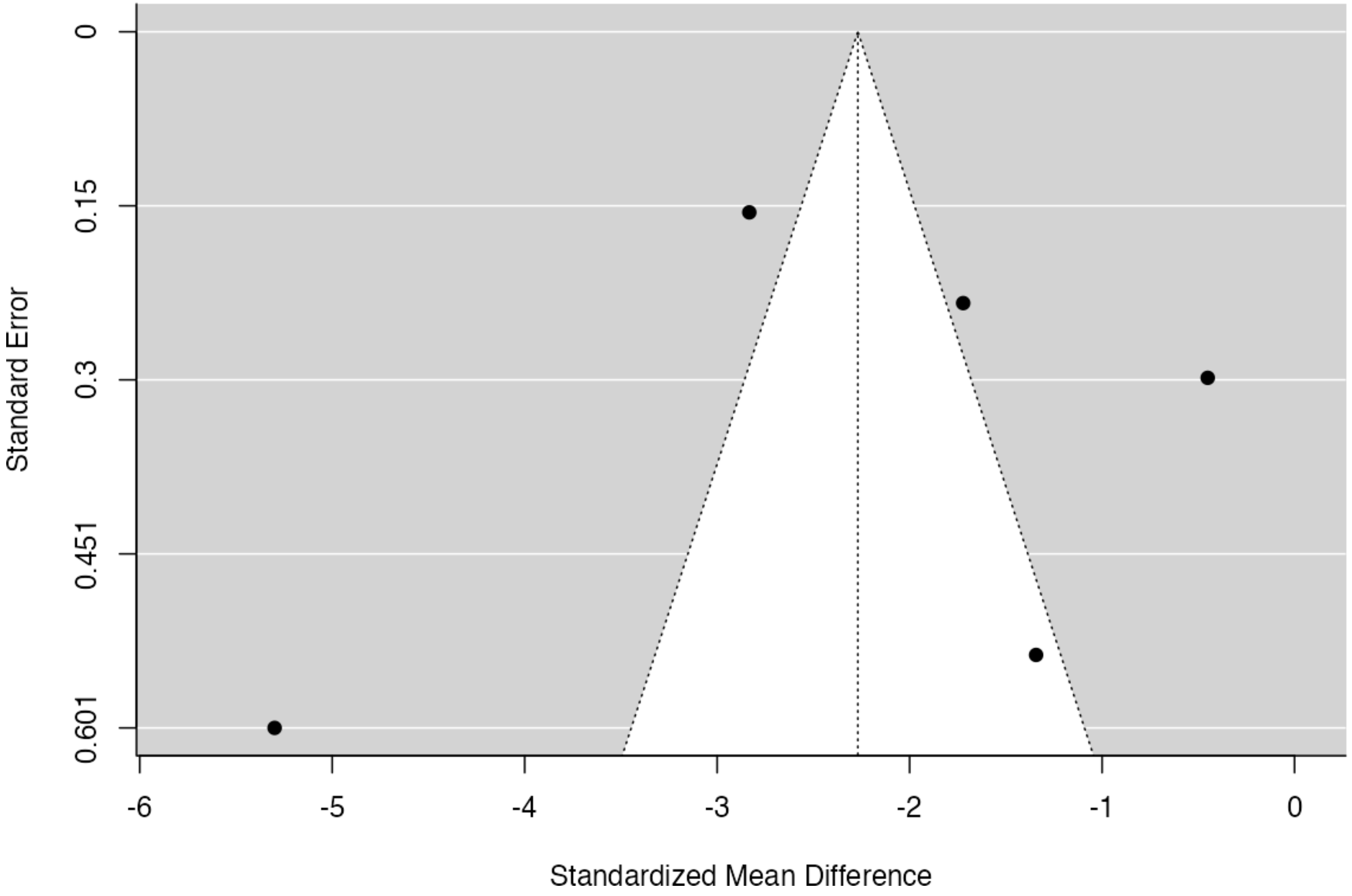

Publication Bias Assessment

Test Name	value	p
Fail-Safe N	539.000	<.001
Begg and Mazumdar Rank Correlation	0.000	1.000
Egger's Regression	–0.988	0.323
Trim and Fill Number of Studies	0.000	.

Nota. Fail-safe N Calculation Using the Rosenthal Approach

Figure S16: Publication bias assessment of studies without targeted sedation on Ramsay score.

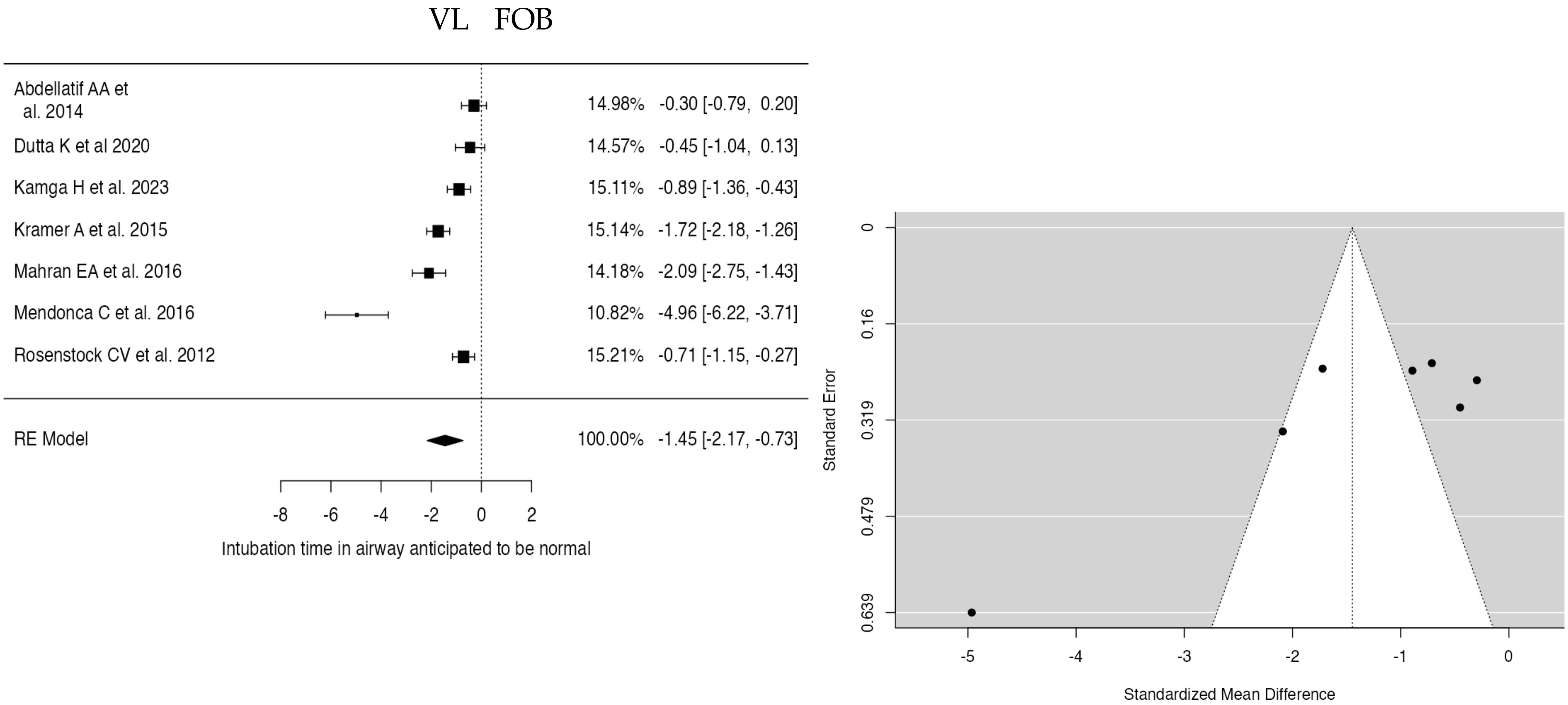

Figure S17: Forest plot for the comparison of intubation time in airway anticipated to be normal.

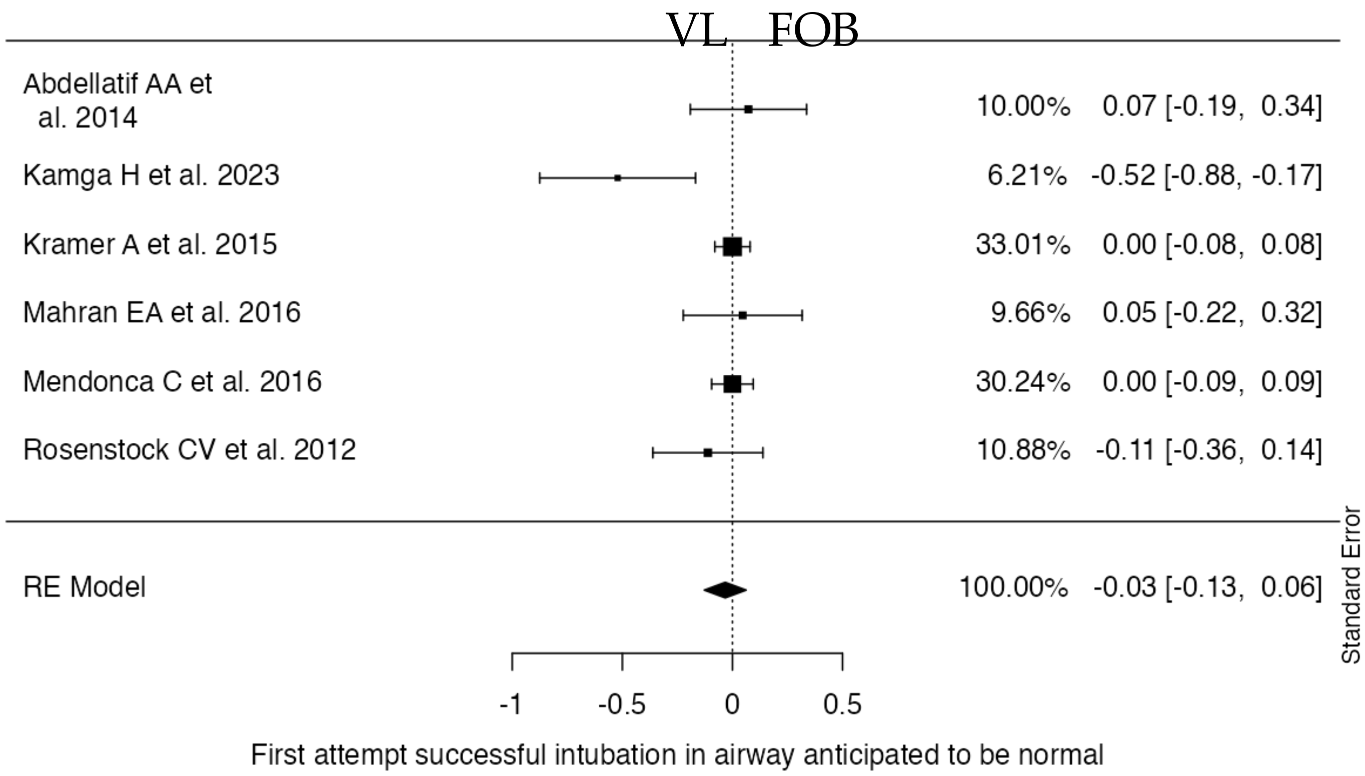

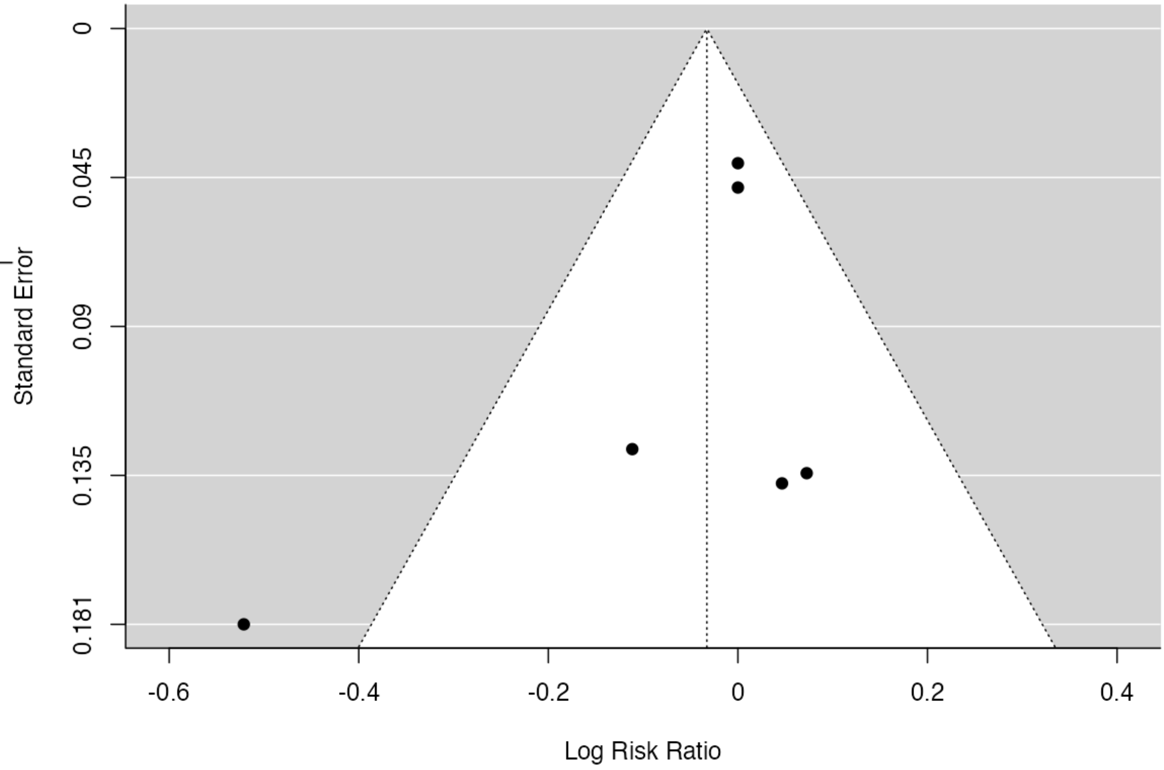

Figure S18: Forest plot for the comparison of first attempt successful intubation in airway anticipated to be normal.

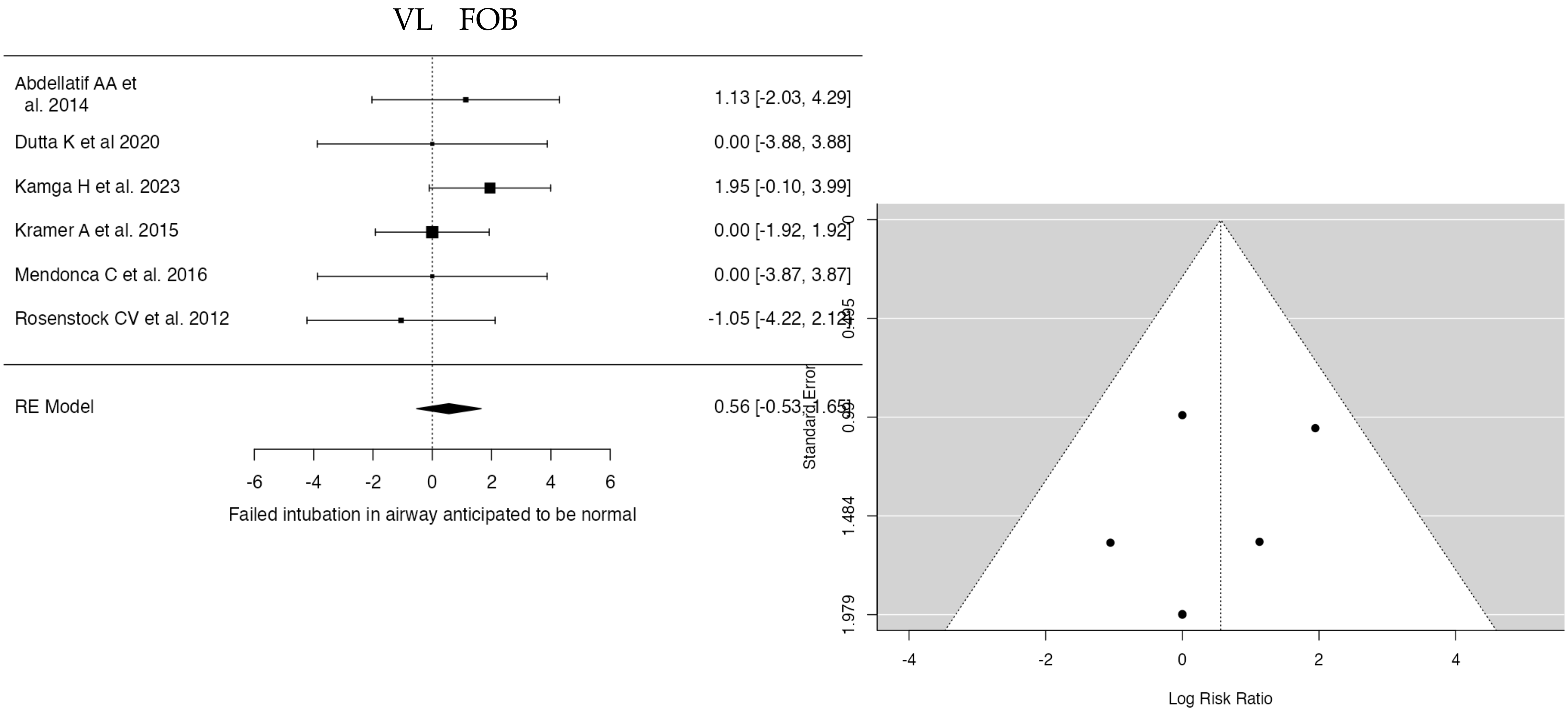

Figure S19: Forest plot for the comparison of failed intubation in airway anticipated to be normal.

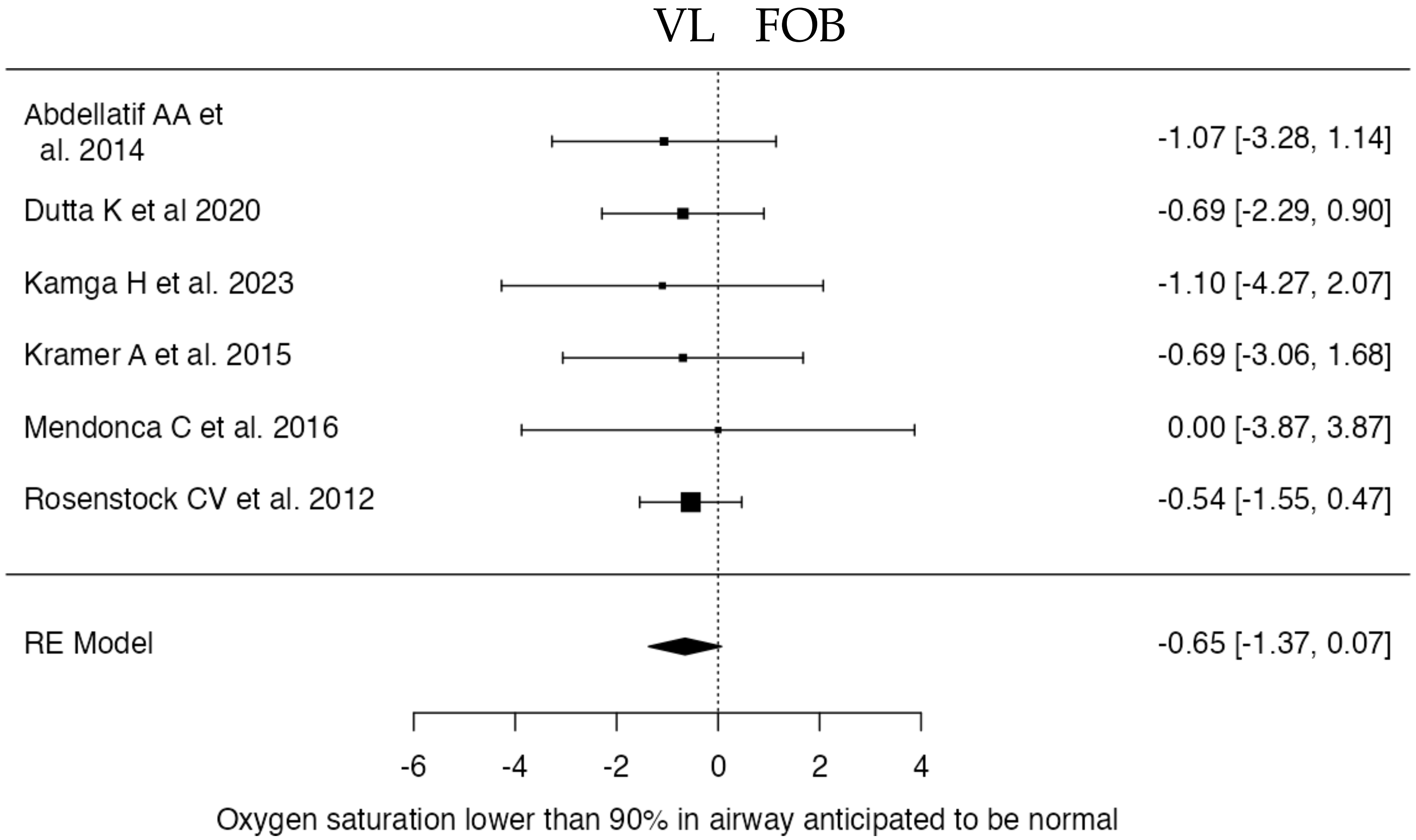

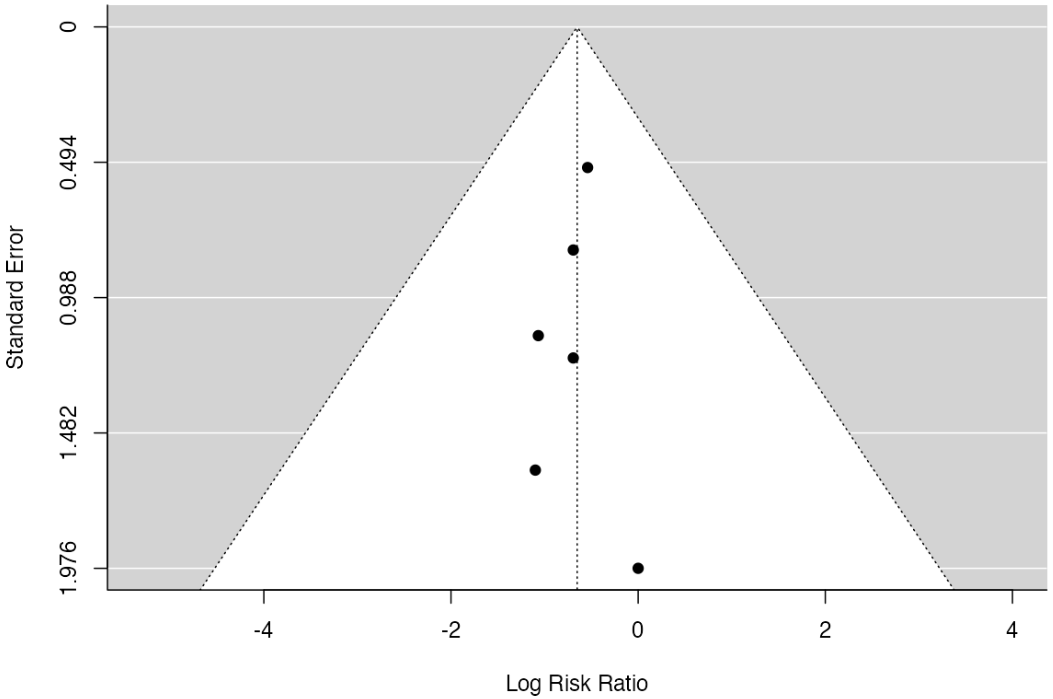

Figure S20: Forest plot for the comparison of oxygen saturation lower than 90% in airway anticipated to be normal.

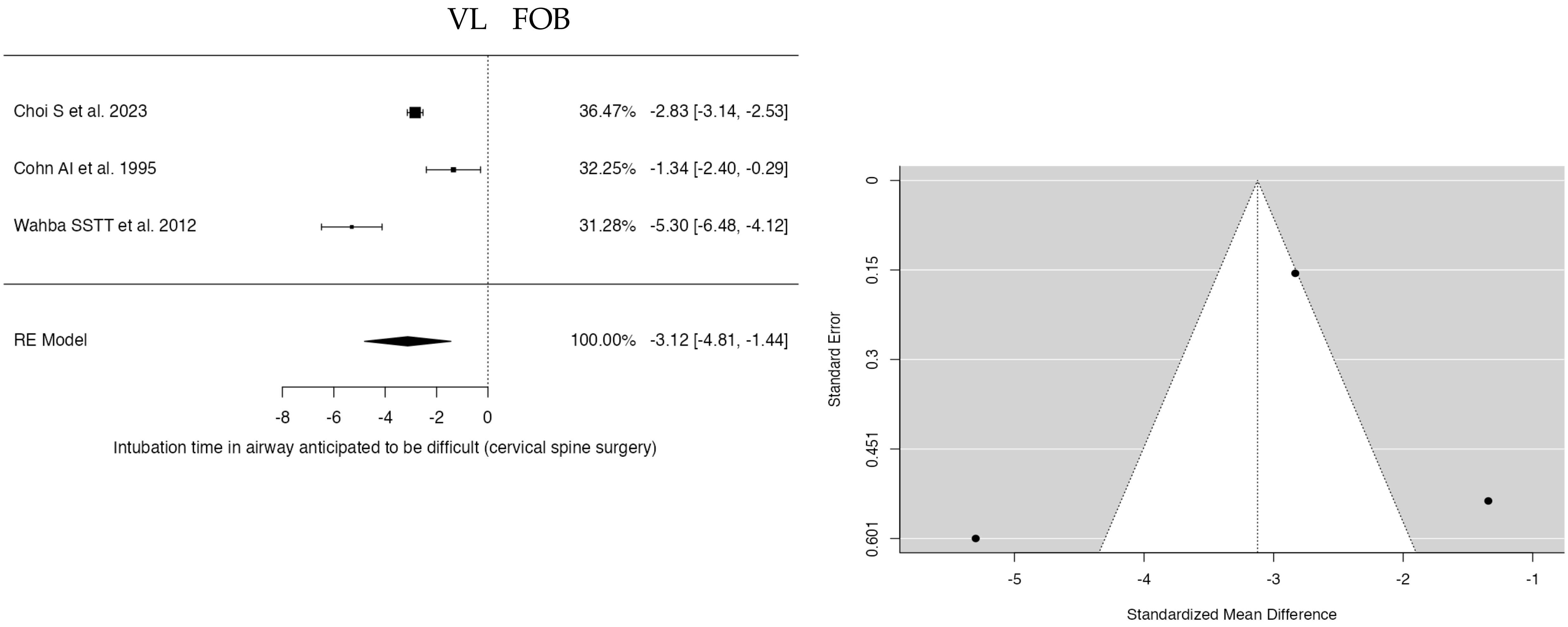

Figure S21: Forest plot for the comparison of intubation time in airway anticipated to be difficult.
